# Single-Atom Ru in CoFe-LDH Drives Efficient Charge Separation on BiVO_4_ for Solar Water Splitting

**DOI:** 10.1007/s40820-025-02062-y

**Published:** 2026-01-19

**Authors:** Wenhui Deng, Gaoshuang He, Haozhi Zhou, Wenhao He, Lei Gan, Chenyu Zhang, Keke Wang, Xiaoqing Qiu, Yang Liu, Wenzhang Li

**Affiliations:** 1 School of Chemistry and Chemical Engineering, Central South University, Changsha, 410083 People’s Republic of China; 2 School of Physical Science and Technology, ShanghaiTech University, Shanghai, 201203 People’s Republic of China; 3 Hunan Provincial Key Laboratory of Chemical Power Sources, Central South University, Changsha, 410083 People’s Republic of China; 4 School of Chemistry and Chemical Engineering, Hunan University of Science and Technology, Xiangtan, 411100 People’s Republic of China

**Keywords:** Photo-electrocatalysis, Water splitting, BiVO_4_ photoanode, Ruthenium single atoms, Layered double hydroxide

## Abstract

Bismuth vanadate (BiVO_4_) is regarded as a promising photoanode for photoelectrochemical (PEC) water splitting. Despite its advantage in band gap and visible-light response, the BiVO_4_ exhibits an unsatisfactory achieving water splitting due to severe charge recombination. Herein, we elucidate an innovative approach involving the incorporation of single Ru atom with a CoFe-LDH cocatalyst (Ru_0.51_-CoFe-LDH) and integrating it onto the BiVO_4_ semiconductor substrate. The resulting Ru_0.51_-CoFe-LDH/BiVO_4_ photoanode film demonstrates commendable charge injection efficiency (76%) and charge collection efficiency (100%). Interestingly, the yield of hydrogen and oxygen increases linearly at a stoichiometric ratio of about 2:1, reaching 158.6 and 67.4 μmol after 140 min of irradiation, respectively. According to experimental characterization and density functional theory calculation, this remarkable performance results from single Ru atoms triggering the electron rearrangement of Ru_0.51_-CoFe-LDH to engineer active sites and optimize interfacial energetics. Additionally, the negative shift of Ru_0.51_-CoFe-LDH band edge gives rise to more conspicuous band bending of the n–n junction formed with BiVO_4_, expediting the separation and transfer of photogenerated electron–hole pairs at the interface. This work furnishes a new preparation perspective for PEC water splitting systems to construct single atoms in the semiconductor substrate.

**Supplementary Information:**

The online version contains supplementary material available at 10.1007/s40820-025-02062-y.

## Highlights

- The single Ru atoms trigger the electron rearrangement of Ru_0.51_-CoFe-LDH to engineer active sites and optimize interfacial energetics.
- The negative shift of Ru_0.51_-CoFe-LDH band edge gives rise to more conspicuous band bending of the n-n junction formed with BiVO_4_.
- The Ru_0.51_-CoFe-LDH/BiVO_4_ photoanode film displays a 3.1 times higher photocurrent density than bare BiVO_4_ and commendable charge collection efficiency (100%).

**Supplementary Information:**

The online version contains supplementary material available at 10.1007/s40820-025-02062-y.

## Graphical abstract

**Figure:**
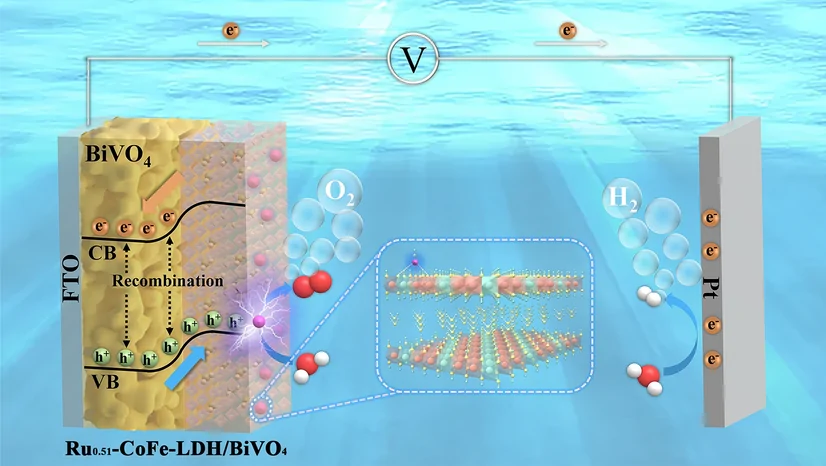

## Introduction

The urgent global transition to clean energy has elevated photoelectrochemical (PEC) water splitting as a front runner for converting solar irradiation into storable hydrogen [1]. Since 1972, Honda and Fujishima first used titanium dioxide to produce hydrogen by photoelectrochemical water splitting [2]. Numerous semiconductor materials (WO_3_ [3], BiVO_4_ [4, 5], BaTaO_2_N [6], etc.) have been developed and applied to photoanodes. Among them, ternary metal oxide BiVO_4_ is regarded as a potential candidate because of its narrow band gap (~ 2.4 eV, high theoretical solar-hydrogen conversion efficiency of 9.2%), suitable band edge position, low cost, and non-toxic. Nevertheless, severe charge recombination and sluggish water oxidation kinetics limit its practical performance [7].

Integrating oxygen‐evolution cocatalysts (OECs) onto BiVO_4_ has emerged as a highly effective mitigation strategy [8, 9]. In particularly, layered double hydroxides (LDHs) offer tunable electronic structures and robust water oxidation activity [10–14]. For instance, Shao et al. demonstrated that NiCo-LDH/BiVO_4_ leverages activated hydroxyl groups to form reactive oxygen species, creating hole-trapping sites for enhanced oxidation [15]. Wang et al. engineered a BiVO_4_ photoanode modified with hollow dodecahedral NiCo-LDH, whose unique structure provided abundant oxygen evolution reaction (OER) active sites and facilitated water adsorption [16]. Despite these advances, precise electronic structure modulation of LDHs active layers on the photoanodes for PEC water splitting remains limited, leaving room for further improvement.

Recent advances in single-atom catalysts (SACs) engineering enabled maximizing metal utilization and tailoring coordination environments to enhance light absorption, intermediate adsorption, and charge transfer [17–19] Among them, noble-metal SACs—particularly Ir and Ru, demonstrate exceptional stability and OER activity, making them promising cocatalysts for photoanode to modulate interfacial charge distribution and electronic structures [20, 21]. However, SACs are thermodynamically prone to aggregation and require supports with abundant unsaturated coordination sites. LDHs, featuring a flexible two-dimensional layered architecture, tunable composition, and high density of surface sites, provide an ideal scaffold for stabilizing isolated noble-metal atoms [22]. Although noble-metal SACs have achieved electrocatalytic OER performance, their integration into PEC systems poses additional complexities [23–25]. In PEC systems, light harvesting, charge separation, and surface catalysis must function cooperatively. Consequently, the role of a cocatalyst extends beyond providing highly active reaction centers; the interfacial band alignment and charge transfer kinetics between the cocatalyst and light absorber become decisive factors in governing overall efficiency. The introduction of single-atom active sites into LDH holds the potential to alter the energy level structure of LDH carriers, thereby contributing to the expansion of LDH applicability. Hence, exploiting the synergistic advantages of LDH supports and SACs presents a promising strategy to advance PEC water splitting performance.

Guided by this rationale, we report a single Ru atom stabilized on CoFe-LDH coated BiVO_4_ films, and engineered active sites and modulated interfacial energetics are achieved to enhance the photoelectrochemical performance of BiVO_4_-based photoanode. Aberration-corrected HAADF-STEM and Fourier-transformed extended X-ray absorption fine structure (EXAFS) spectra confirmed uniform Ru dispersion in CoFe-LDH. Density functional theory calculations revealed that the introduction of single Ru atom into CoFe-LDH can trigger the electron rearrangement of Ru_0.51_-CoFe-LDH to optimize the binding energy between the active site and intermediates. The resulting Ru_0.51_-CoFe-LDH photoanode achieved 4.51 mA cm^−2^ at 1.23 V vs. RHE under AM 1.5G, with 76% charge injection efficiency and stable operation over 10 h. This LDH-SACs coupling strategy provides a versatile platform for advancing PEC water splitting via engineering actives and modulating interfacial energetics.

## Experimental Section

### Chemicals

Cobalt(II) nitrate hexahydrate (Co(NO_3_)_2_·6H_2_O), iron(III) nitrate nonahydrate (Fe(NO_3_)_3_·9H_2_O), sodium hydroxide (NaOH), sodium carbonate (Na_2_CO_3_), ruthenium chloride (RuCl_3_·xH_2_O), bismuth nitrate pentahydrate (Bi(NO_3_)_3_·_5_H_2_O, 98%), potassium iodide (KI, 95%), nitric acid (HNO_3_, 69%), p-benzoquinone (98.0%), ethanol (99.7%, GR), vanadyl acetyl-acetonate (VO(acac)_2_), acetone (GR), dimethyl sulfoxide (DMSO, 99.9%), and boric acid (H_3_BO_3_, 99.5%) were purchased from Sinopharm Chemical Reagent Co, Ltd. All the reagents were analytical grade and were used directly without further purification.

### Preparation of CoFe-LDH Material

CoFe-LDH nanosheets were synthesized using co-precipitation method. Firstly, prepare 40 mL of metal salt solution A (Co: Fe = 2:1) and 40 mL of alkaline environmental solution B (0.318 g Na_2_CO_3_ and 0.84 g NaOH), respectively. Then slowly added solution A and solution B simultaneously to a beaker containing 80 mL of deionized water. The mixed solution was stirred at room temperature for 24 h to gradually form a yellow brown solid precipitate, which was collected by centrifugation and washed three times with ethanol and water. The resulting sample was dried in a vacuum oven at 60 ℃ for 18 h and named CoFe-LDH nanosheets.

### Preparation of Rux-CoFe-LDH Photoanodes

Synthesis of Ru_x_-CoFe-LDH nanosheets. Dissolve different amounts of RuCl_3_·xH_2_O (2, 5, and 20 mg) in a flask containing 40 mL NaOH solution (0.01 M). Then, 500 mg CoFe-LDH nanosheets were added to the solution, ultrasound for 15 min, and then stirred at room temperature for 12 h. The gray solids were collected by centrifugation and washed three times with ethanol and water. The collected samples were dried in a vacuum oven at 60 °C for 18 h. The anchored ruthenium content was determined by inductively coupled plasma optical emission spectrometry (ICP–OES) to be 0.15, 0.51, and 1.52 wt%, respectively. The samples were named Ru_0.15_-CoFe-LDH, Ru_0.51_-CoFe-LDH, and Ru_1.52_-CoFe-LDH according to the different ruthenium content.

### Preparation of CoFe-LDH/BiVO_4_ and Ru_x_-CoFe-LDH/BiVO_4_ Photoanodes

The BiVO_4_ photoanode thin film was synthesized following previously reported procedures [26]. First, 3.32 g of KI was dissolved in 50 mL of deionized water, and the pH of the solution was adjusted to 1.7 using nitric acid. Subsequently, 0.9701 g of Bi(NO_3_)_3_·5H_2_O was added gradually under vigorous stirring until fully dissolved, yielding Solution A. Meanwhile, 0.52 g of p-benzoquinone was dissolved in 20 mL of anhydrous ethanol to form Solution B. Solution B was then slowly introduced to Solution A to generate the electrolyte for BiOI electrodeposition. Electrodeposition was operated at − 0.1 V vs. Ag/AgCl for 300 s, resulting in the formation of a reddish-brown BiOI film on the FTO surface. Next, 150 μL of a DMSO solution containing VO(acac)_2_ (0.2 mol L^−1^) was drop-cast onto the BiOI film in air, followed by thermal treatment in air at 450 °C for 2 h with a heating rate of 2 ℃ min^−1^ to obtain crude BiVO_4_. The excess V_2_O_5_ in the crude BiVO_4_ was removed by immersing the film in 1.0 M NaOH under stirring, after which the film was rinsed thoroughly with ethanol and DI water. Due to the surface potential of LDH was opposite to that of BiVO_4_ (Fig. S1 and Table S2), CoFe-LDH/BiVO_4_, Ru_0.15_-CoFe-LDH/BiVO_4_, Ru_0.51_-CoFe-LDH/BiVO_4_, and Ru_1.52_-CoFe-LDH/BiVO_4_ photoanodes were formed through a multi-purpose coupling strategy. The synthesized LDH was dispersed in ethanol solution to form a dispersion solution with a concentration of 1 mg mL^−1^, and then dropped onto the surface of BiVO_4_ film. The dispersed solution was spin coated at a speed of 1000 rpm for 30 s and dried on a heating plate at 70 ℃ for 5 min and repeated three times.

### Characterizations, Photoelectrochemical Measurements, and Computational Details

Characterization, Photoelectrochemical measurements, and Computational details could be found in the Supplementary Materials.

## Results and Discussion

### Chemical Structure Characterization of Ru_0.51_-CoFe-LDH Nanosheets

**Scheme 1:**
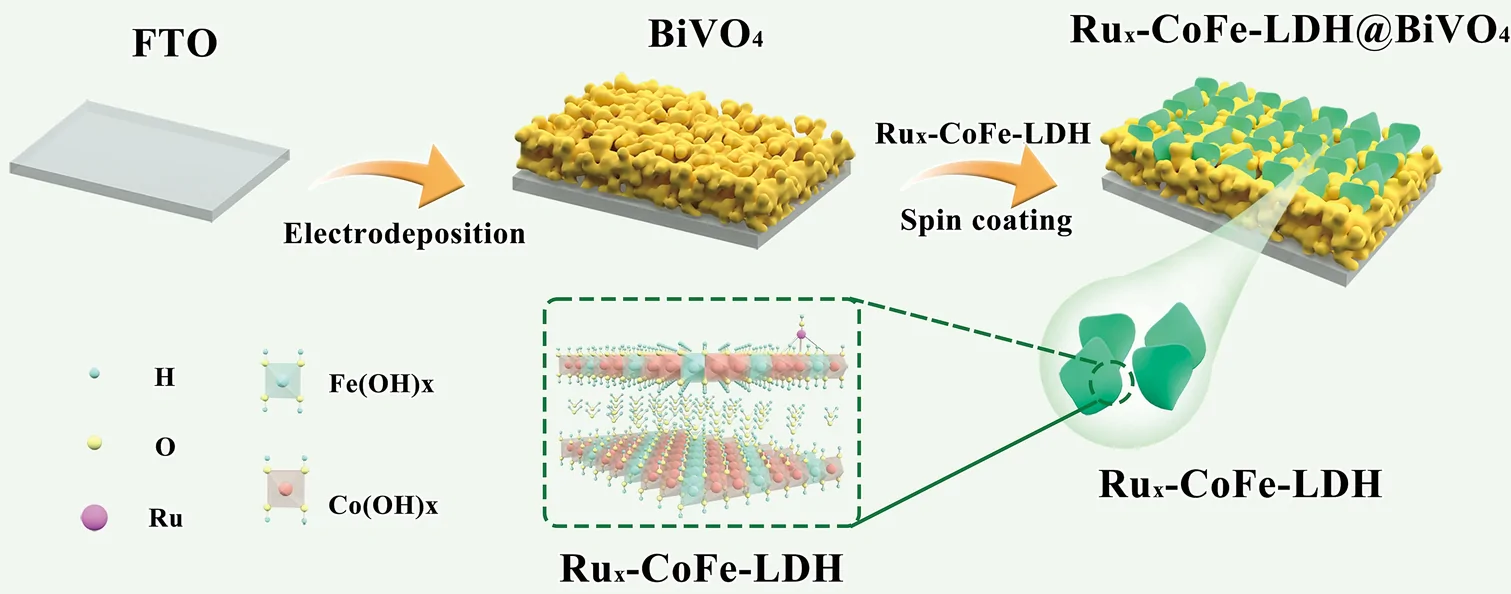
Schematic illustration of synthesis of Ru_0.51_-CoFe-LDH/BiVO_4_

**Fig. 1:**
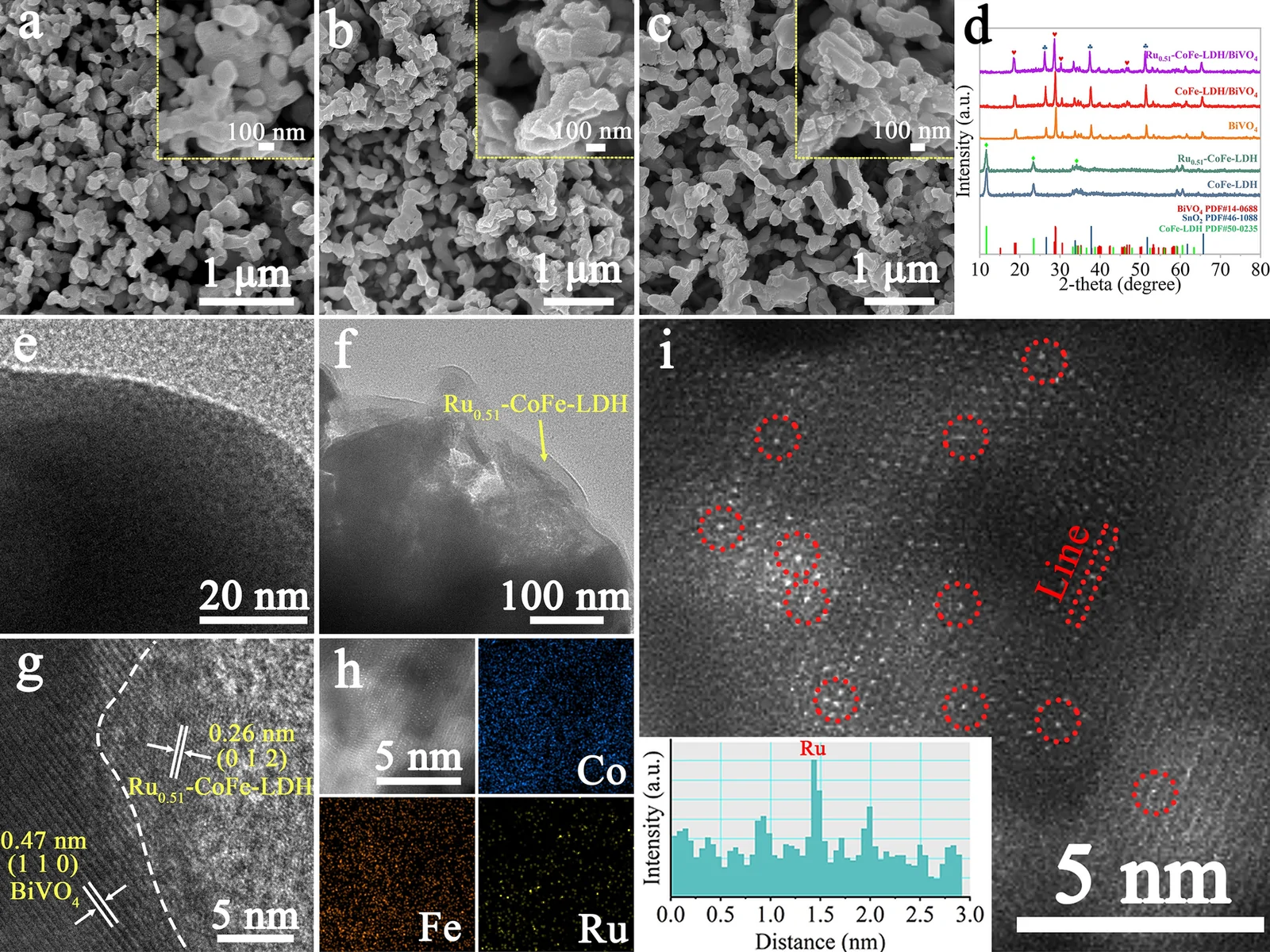
**a**–**c** Top-view SEM images of BiVO_4_, CoFe-LDH/BiVO_4_ and Ru_0.51_-CoFe-LDH/BiVO_4_. **d** XRD patterns, **e–f** TEM of BiVO_4_ and Ru_0.51_-CoFe-LDH/BiVO_4_. **g** HR-TEM of Ru_0.51_-CoFe-LDH/BiVO_4_. **h** EDX images of Ru_0.51_-CoFe-LDH, and **i** AC-HAADF-TEM images of Ru_0.51_-CoFe-LDH, insert: the line intensity distribution profiles along the middle of dotted box tagged area

The Ru atomic active sites were successfully anchored on the CoFe-LDH nanosheets by the deposition–precipitation method (Experimental Section for details). The ruthenium content of Ru_0.51_-CoFe-LDH nanosheets is 0.51 wt% determined by ICP-OES. The Ru_0.51_-CoFe-LDH/BiVO_4_ integrated photoanode was synthesized by integrating the dispersed Ru_0.51_-CoFe-LDH nanosheets on the surface of the wormlike shape BiVO_4_ array (Scheme 1). Scanning electron microscopy (SEM) shows the slightly rough surface for Ru_0.51_-CoFe-LDH/BiVO_4_ and CoFe-LDH/BiVO_4_ photoanode (Fig. 1a–c), which is different from the bare BiVO_4_ photoanode with smooth surface. The cross-sectional images (Fig. S2) demonstrate relatively uniform thickness of the three samples (about 1.3 μm). In Fig. 1d, it can be seen that the diffraction peaks at 11.6°, 23.4°, and 34.1° correspond to the (003), (006), and (012) facets of CoFe-LDH (JCPDS 50-0235), respectively. There is no characteristic peak of the new phase after loading a small amount of Ru atoms, which can be explained that the trace Ru atoms anchored on LDH.

The XRD patterns of the thin film arrays grown on FTO substrates show that the diffraction peaks correspond to the monoclinic BiVO_4_ phase (JCPDS 83-1688) and SnO_2_ (JCPDS PDF 41-1445). Notably, there is no significant change between BiVO_4_ and Ru_0.51_-CoFe-LDH/BiVO_4_, which may be explained by the low content of LDH coated on the surface. Transmission electron microscopy (TEM) images display that Ru_0.51_-CoFe-LDH adheres to BiVO_4_ tightly (Fig. 1e, f). Furthermore, HR-TEM images show that the lattice spacing of 0.47 and 0.26 nm correspond to the (110) crystal facet of BiVO_4_ and the (012) crystal facet of Ru_0.51_-CoFe-LDH, respectively. (Fig. 1g). Energy-dispersive X-ray spectroscopy confirms the presence of Bi, V, O, Ru, Co, and Fe, with these elements uniformly distribute throughout the architecture (Figs. 1h and S3). More importantly, the AC-HAADF-STEM images of Ru_0.51_-CoFe-LDH reveal the atomically dispersion of Ru species in CoFe-LDH (marked by red circles). The intensity distribution profiles of the dotted box tagged area further illustrate that the single Ru atoms are significantly brighter than other atoms (insert of Fig. 1i).

**Fig. 2:**
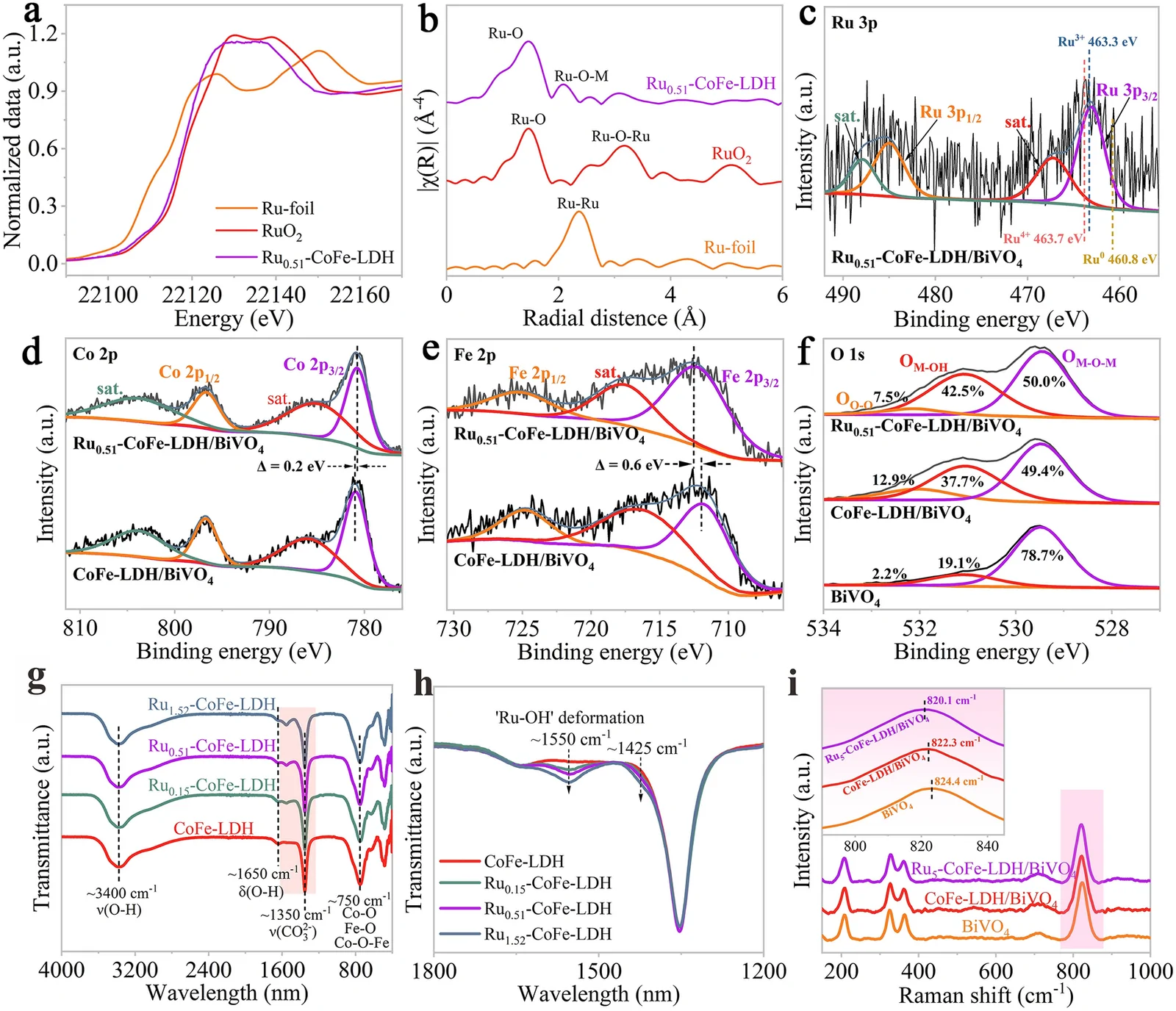
**a** XANES spectra. **b** R-space Ru K-edge EXAFS spectra. **c** XPS spectra of Ru 3*p* in Ru_0.51_-CoFe-LDH/BiVO_4_. **d**-**e** XPS spectra of Co 2*p* and Fe 2*p* in Ru_0.51_-CoFe-LDH/BiVO_4_ and CoFe-LDH/BiVO_4_. **f** XPS spectra of O 1*s* in three photoanodes. **g** FT-IR absorption spectra of different LDH. **h** partial enlargement of the selective area in **g**. **i** Raman spectra of three photoanodes

To further elucidate the structural influence of incorporating atomically dispersed Ru sites into the CoFe-LDH, X-ray absorption fine structure (XAFS) spectroscopy was employed to reveal detailed structural and coordination environments. The Ru K-edge X-ray absorption near-edge structure (XANES) shows that Ru_0.51_-CoFe-LDH lies between metallic Ru and RuO_2_ (Fig. 2a). Generally, there is an approximate linear relationship between absorption edge energy and the calculated oxidation state. Given the approximate linear correlation between absorption edge energy and oxidation state, linear fitting indicates that the Ru species in Ru_0.51_-CoFe-LDH possess an average oxidation state of + 2.1 (Fig. S4a). The local structure of Ru_0.51_-CoFe-LDH was further investigated using the Fourier-transformed extended X-ray absorption fine structure (EXAFS) spectra (Fig. 2b). Compared with reference samples (Ru foil, RuO_2_), the Ru_0.51_-CoFe-LDH sample displays no discernible Ru–Ru or Ru–O–Ru coordination peaks associated with aggregated or clustered Ru species. Only a dominant Ru–O bond in the first shell and a weak Ru–O-M bond (M = Co or Fe) at higher energy levels are observed. Quantitative EXAFS fitting (Fig. S4b, c) reveals that the Ru–O coordination number is approximately four (Table S4), consistent with a fourfold-coordinated Ru–O configuration located at the surface of the CoFe-LDH lattice. These findings unequivocally confirm the presence of atomically dispersed Ru on the Ru_0.51_-CoFe-LDH support.

The chemical composition and electronic properties of three photoanodes are discerned through X-ray photoelectron spectroscopy (XPS), revealing the distinct presence of Ru, Co, Fe, O, Bi, and V elements across the entire spectrum range of 0–1200 eV (Fig. S5). As shown in Fig. 2c, the binding energy peaks at 463.1 and 485 eV are attributed to the 2*p*_3/2_ and 2*p*_1/2_ orbitals of Ru, respectively, indicating that the existence of Ru is a special valence state between 0 and + 3 valences [27] At 467.3 and 487.8 eV, they correspond to the accompanying satellite peaks of Ru [28]. Compared with CoFe-LDH/BiVO_4_, the binding energy of the Co 2*p* orbitals of Ru_0.51_-CoFe-LDH/BiVO_4_ shifted negatively by ~ 0.2 eV, indicating a slight electron-rich state at the Co site (Fig. 2d) [23] Similarly, a positive shift of ~ 0.6 eV in the binding energy of the Fe 2*p* orbitals of Ru_0.51_-CoFe-LDH/BiVO_4_ indicates that the Fe site also indicates electron-deficient states (Fig. 2e). The changes in the electronic structure of the main metal Co and Fe in the cocatalyst can be attributed to the introduction of the noble-metal Ru^δ+^ (0 < δ < 3) with strong electron withdrawing ability, which allow more electrons to be transferred to the Ru active site through Ru–O–M (M = Co or Fe) bonds, thereby making the electron rearrangement of Ru_0.51_-CoFe-LDH [23]. Figure 2f shows that the asymmetric peaks of O 1*s* are deconvoluted by Gaussian fitting at 529.5, 531.1, and 532.1 eV, respectively attributed to metal oxides (M–O), metal hydroxides (M-OH), and surface adsorbed oxygen [29]. Among them, due to the loading of LDH on the surface of BiVO_4_ photoanode, the content of M-OH increases significantly. Meanwhile, the introduction of Ru atoms further increases the proportion of M-OH, which may be explained by its anchoring on the surface of CoFe-LDH in the form of Ru–OH (Table S3). In addition, Fig. S6 indicates that there is no significant difference in the core level XPS spectral peak positions of Bi 4*f* and V 2*p* among the three photoanodes [30].

Fourier transform infrared (FTIR) spectroscopy was employed to investigate the impact of the introduction of Ru atomic active sites on the bonding structure of CoFe-LDH effectively. Figure 2g illustrates the FT-IR spectra of CoFe-LDH with different Ru contents, showing minimal changes in the absorption peaks of most groups. Importantly, the introduction of ruthenium gradually leads to the emergence of a "Ru-OH" induced deformation vibration absorption band in LDH at approximately 1550 cm^−1^, with the peak intensity increasing with the augmentation of Ru sites (Fig. 2h) [31], which is consistent with the XPS results. Similarly, the characteristic bond vibration translation modes of the three photoanode films are also analyzed through FT-IR (Fig. S7). No significant difference is observed in the infrared spectra between the BiVO_4_ photoanode loaded with LDH and the bare BiVO_4_ photoanode, possibly due to the relatively low loading amount of LDH. Further investigation was conducted using Raman spectroscopy to investigate the effect of LDH introduction on the structure of BiVO_4_ (Fig. 2i). Compared to the bare BiVO_4_ (824.4 cm^−1^), the V–O bond symmetric stretching mode of Ru_0.51_-CoFe-LDH/BiVO_4_ and CoFe-LDH/BiVO_4_ photoanodes shifted slightly to 820.1 and 822.3 cm^−1^, respectively [32]. According to formula S2, the length of the V–O bond increase to 1.6997 Å from 1.6983 Å due to the existence of Ru, indicating that there is a strong interaction between single-atom Ru and the substrate.

**Fig. 3:**
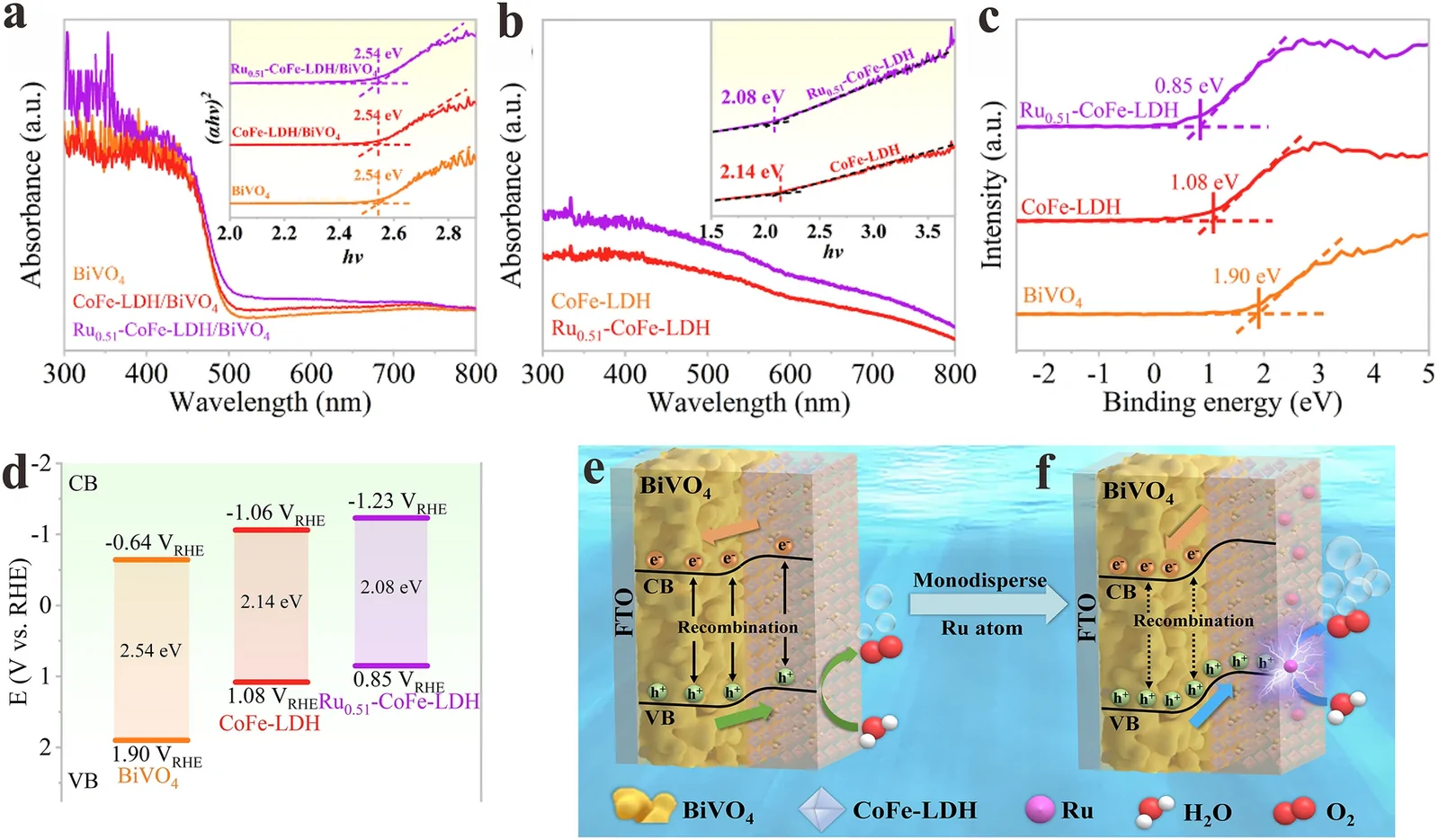
**a** UV–Vis DRS spectra of three photoanodes; insert: Tauc plots. **b** UV–Vis DRS spectra of CoFe-LDH and Ru_0.51_-CoFe-LDH; insert: Tauc plots. **c** VB-XPS spectra of three photoanodes. **d** Band structure of BiVO_4_, CoFe-LDH and Ru_0.51_-CoFe-LDH. Schematic diagrams of the charge transfer process for **e** CoFe-LDH/BiVO_4_ and **f** Ru_0.51_-CoFe-LDH/BiVO_4_ photoanodes

To elucidate the effect of the introduction of Ru sites on the energy level structure between LDH and BiVO_4_ photoanodes, the bandgap and band edge position of the samples were analyzed using UV–Visible diffuse reflectance spectroscopy (UV–Vis DRS) and valence band XPS (VB-XPS). As shown in Fig. 3a, all three photoanodes exhibit comparable optical absorption ranges. Notably, Ru_0.51_-CoFe-LDH/BiVO_4_ displays a modest enhancement in absorption within the 550–650 nm region, consistent with the absorption behavior of Ru_0.51_-CoFe-LDH and CoFe-LDH (Fig. 3b). VB-XPS analysis (Fig. 3c) reveals valence band (VB) edge positions of 1.90, 1.08, and 0.85 eV for BiVO_4_, CoFe-LDH, and Ru_0.51_-CoFe-LDH, respectively.

The bandgaps, estimated from the linear regions of the Tauc plots, were determined to be 2.54, 2.14, and 2.08 eV for BiVO_4_, CoFe-LDH, and Ru_0.51_-CoFe-LDH, respectively. Accordingly, the conduction band (CB) edge positions, calculated using Eq. S4, are − 0.64, − 1.06, and − 1.23 eV, respectively [33]. The upward shift of the VB edge in Ru_0.51_-CoFe-LDH induces greater band bending at the n–n heterojunction formed with BiVO_4_, thereby increasing the driving force for interfacial charge separation and facilitating photogenerated hole transfer (Fig. 3d). To elucidate the role of single Ru atom in the PEC water splitting process, Fig. 3e, f illustrates the proposed reaction mechanism of the Ru_0.51_-CoFe-LDH/BiVO_4_ photoanode. For pristine BiVO_4_, the relatively large charge transfer resistance and sluggish surface reaction kinetics limit PEC performance. For the CoFe-LDH/BiVO_4_ photoanode, the heterojunction-induced band bending enhances charge transfer efficiency (insert of Fig. 3e). The electronic rearrangement induced by Ru–O bonds in CoFe-LDH leads to an optimized band structure. Consequently, the interface between Ru_0.51_-CoFe-LDH and BiVO_4_ exhibits more pronounced band bending and a stronger built-in electric field, which facilitates the separation and injection of photogenerated holes from the BiVO_4_ bulk to the Ru_0.51_-CoFe-LDH interface, leading to substantially improved PEC water oxidation performance (insert of Fig. 3f).

### PEC Water Splitting Performance of Ru_0.51_-CoFe-LDH Nanosheets

**Fig. 4:**
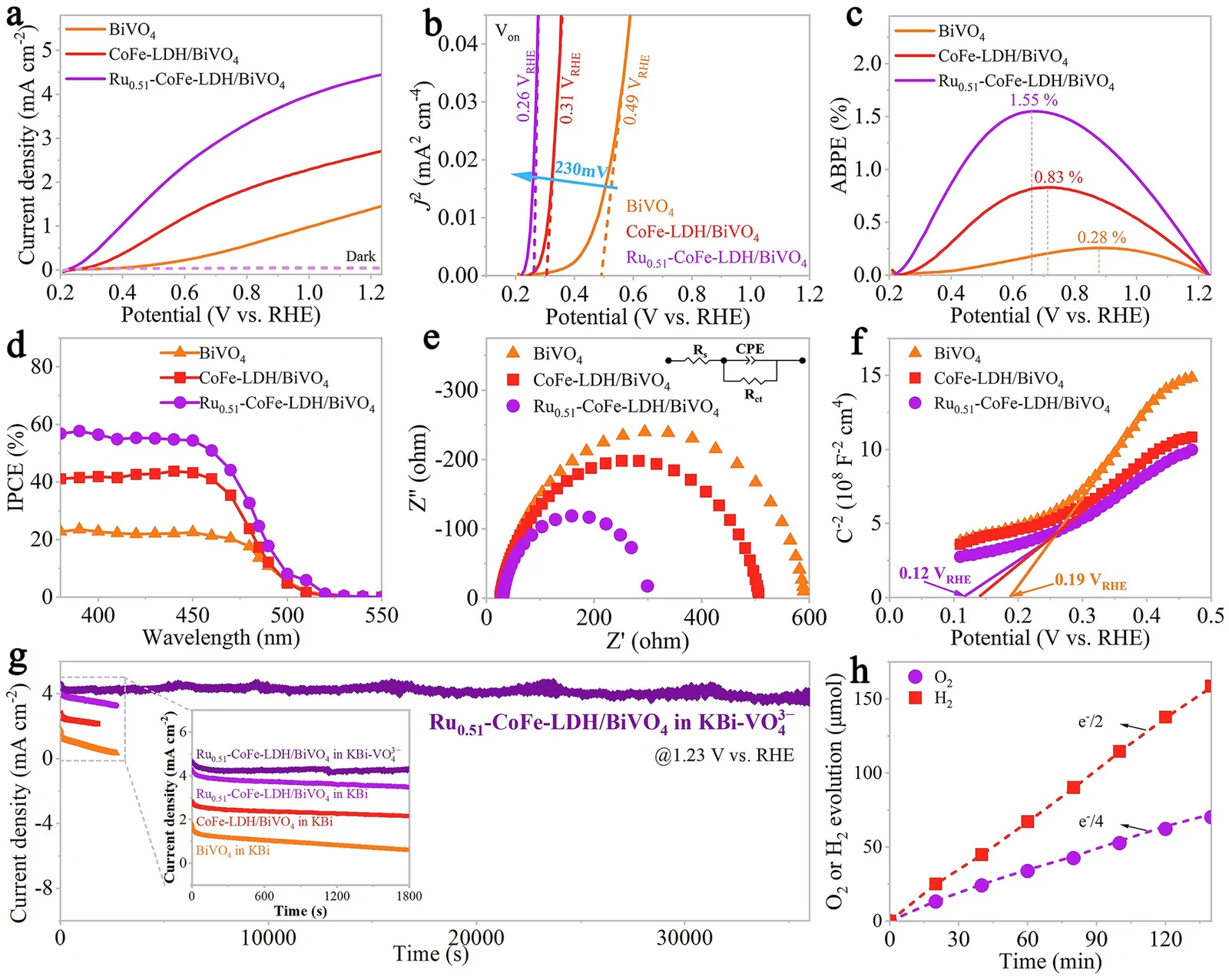
**a** LSV curves, **b** Onset potential, **c** ABPE curves, **d** IPCE curves, **e** PEIS plots, **f** Mott-Schottky plots of three photoanodes. **g** Stability tests measured in 0.5 M KBi or KBi-VO_4_^3−^ buffer solution (pH = 9.2) at 1.23 V vs. RHE. **h** H_2_ and O_2_ gases evolution curves of Ru_0.51_-CoFe-LDH/BiVO_4_ photoanode

The PEC water splitting performance of Ru_0.51_-CoFe-LDH/BiVO_4_, CoFe-LDH/BiVO_4_ and BiVO_4_ photoanodes was evaluated by linear sweep voltammetry (LSV) curves in a 0.2 M KH_2_PO_4_/K_2_HPO_4_ buffer solution (KPi, pH = 7) under at AM 1.5G illumination (~ 100 mW cm^−2^). As shown in Fig. 4a, the Ru_0.51_-CoFe-LDH/BiVO_4_ photoanode manifests the highest performance, achieving a photocurrent density of 4.51 mA cm^−2^ at 1.23 V vs. RHE, demonstrating a value 3.1 times higher than that of bare BiVO_4_ and outperforming most reported BiVO_4_-based photoanodes (Fig. S8 and Table S5). The inferior performance of Ru_0.15_-CoFe-LDH/BiVO_4_ arises from an insufficient density of catalytically active sites at low Ru loading. To elucidate the decline in PEC activity at higher Ru contents, the microstructures of Ru_0.51_-CoFe-LDH/BiVO_4_ and Ru_1.52_-CoFe-LDH/BiVO_4_ were examined by TEM (Fig. S9). The Ru_1.52_-CoFe-LDH/BiVO_4_ sample, with higher Ru loading, exhibits nanoparticle-like clusters or aggregates on the surface, which lead to the degradation in PEC performance. Therefore, the Ru_0.51_-CoFe-LDH/BiVO_4_ was selected for subsequent structural characterization and performance evaluation. Compared to BiVO_4_ (0.49 V_RHE_), the onset potentials of CoFe-LDH/BiVO_4_ (0.31 V_RHE_) and Ru_0.51_-CoFe-LDH/BiVO_4_ (0.26 V_RHE_) shift negatively by 180 and 230 mV, respectively (Fig. 4b). Ru_0.51_-CoFe-LDH/BiVO_4_ also presents smaller onset potentials than the reported values of LDH modified BiVO_4_ films (CoMn-LDH/BiVO_4_(0.31 V_RHE_) [34], SAs Pt/AC-CoFe/BiVO_4_ (0.35 V_RHE_) [22], and NiFeY-LDH/BiVO_4_(0.31 V_RHE_) [35]. It means that the incorporation of atomically dispersed Ru sites lowers the potential barrier for water oxidation and accelerates its reaction kinetics [36]. The Ru_0.51_-CoFe-LDH/BiVO_4_ photoanode also achieved a maximum applied bias photon-to-current efficiency (ABPE) of 1.55%, which is 5.3 times that of pristine BiVO_4_ and 1.9 times that of CoFe-LDH/BiVO_4_ (Fig. 4c).

The incident photon-to-current conversion efficiency (IPCE) reveal similar optical absorption ranges for all three photoanodes, with a cutoff wavelength of ~ 515 nm (Fig. 4d). Across the visible spectrum, the IPCE follows the order: Ru_0.51_-CoFe-LDH/BiVO_4_ > CoFe-LDH/BiVO_4_ > BiVO_4_. Furthermore, the photoelectrochemical impedance spectroscopy (PEIS) was employed to investigate interfacial charge transfer dynamics. As shown in Fig. 4e, the Nyquist diagram of Ru_0.51_-CoFe-LDH/BiVO_4_ photoanode exhibits the smallest semicircle radius, corresponding to the lowest charge transfer resistance. The charge transfer resistance of the three sample photoanodes were quantified by fitting the impedance spectra. The values obtained are 272.7, 481.6, and 564.8 Ω, respectively (Table S6), confirming that Ru_0.51_-CoFe-LDH/BiVO_4_ exhibits the lowest charge transfer resistance. This suggests that the charge transfer process during the water oxidation reaction occurs more rapidly at the photoanode/electrolyte interface for Ru_0.51_-CoFe-LDH/BiVO_4_. Figure 4f illustrates positive slopes for Mott-Schottky (M-S) curves slopes of the three photoanodes at 1 kHz, indicating n-type semiconducting behavior. The flat band potential (*E*_fb_) of Ru_0.51_-CoFe-LDH/BiVO_4_ shifts from 0.19 V_RHE_ (BiVO_4_) to 0.12 V_RHE_, consistent with the trend observed for the onset potential. This shift suggests a reduction in the Fermi level pinning effect at the photoanode surface [37]. The long-term stability of photoanode films is a critical factor for their practical PEC applications. The inset of Fig. 4g illustrates a continuous decrease in photocurrent densities for both CoFe-LDH/BiVO_4_ and BiVO_4_, whereas Ru_0.51_-CoFe-LDH/BiVO_4_ present a much slower decline. Within 30 min, the retention rate is 32%, 76%, and 81% for BiVO_4_, CoFe-LDH/BiVO_4_ and Ru_0.51_-CoFe-LDH/BiVO_4_, respectively. After the long-term stability test, the morphology of the Ru_0.51_-CoFe-LDH/BiVO_4_ remained completely unchanged. Moreover, there is no change in the peak strength and location of the Ru_0.51_-CoFe-LDH/BiVO_4_ for XRD and Raman spectra (Figs. S10 and S11). The introduction of Ru atomic sites alleviates the decline in photocurrent density, likely by promoting the rapid consumption of photogenerated carriers and reducing the formation of surface defect states. However, the stability issue is not completely resolved. The instability of BiVO_4_ photoanodes is primarily due to the formation of a BiO_x_ layer from VO_4_^3−^ dissolution at high bias voltages [38]. To mitigate VO_4_^3−^ dissolution, 0.1 M NaVO_3_ is added to react with OH⁻ and form VO_4_^3−^ (KBi- VO_4_^3−^), maintaining the KBi electrolyte’s concentration and pH. The reaction equation is as follows [39]:

$${\mathrm{VO}}_{3}^{ - } {\text{ + 2OH}}^{ - } { = \mathrm{VO}}_{4}^{3 - } {\text{ + H}}_{{2}} {\mathrm{O}}$$

$${\mathrm{BiVO}}_{{4}} { = \mathrm{VO}}_{4}^{3 - } {\text{ + Bi}}^{3 + }$$

Notably, the Ru_0.51_-CoFe-LDH/BiVO_4_ photoanode retains stability for over ten hours in the KBi-VO_4_^3−^ electrolyte at 1.23 V vs. RHE. Hydrogen and oxygen production increase linearly with irradiation time at a stoichiometric ratio of ~ 2:1, reaching 158.6 and 67.4 μmol after 140 min, with Faraday efficiencies of ~ 100% and ~ 93%, res (Fig. 4h).

**Fig. 5:**
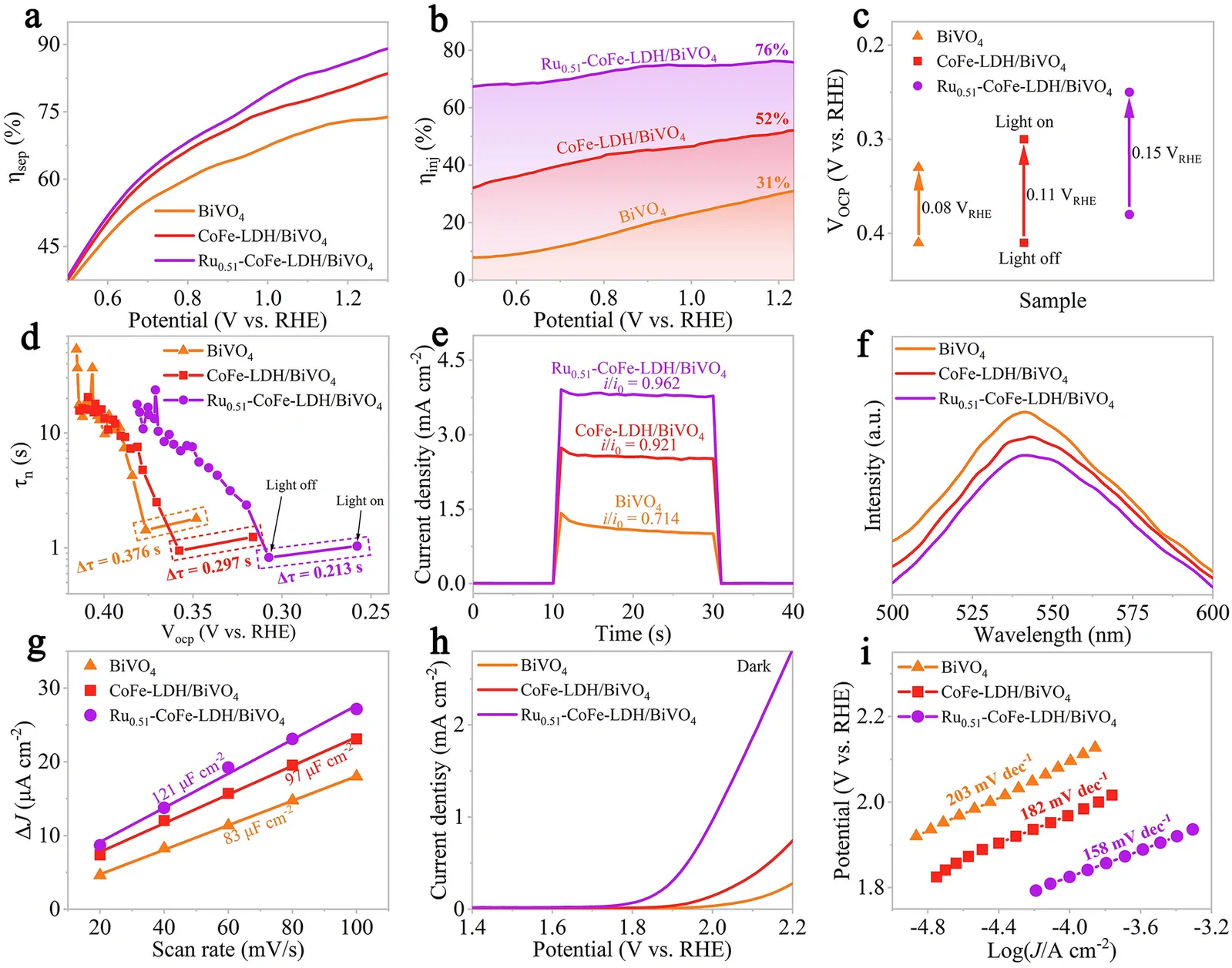
**a** Charge separation efficiency, **b** charge injection efficiency, **c** OCP values. **d** OCP-derived carrier transfer lifetimes, **e** transient photocurrent curves, **f** Steady-state PL spectra, **g** linear fitting of current density and scan rate, **h** polarization curves measured in dark for OER, **i** Tafel plots of three photoanodes

To elucidate the mechanisms behind enhanced PEC water splitting, the optoelectrical properties, electrochemical behavior, and charge transfer dynamics of the BiVO_4_, CoFe-LDH/BiVO_4_, and Ru_0.51_-CoFe-LDH/BiVO_4_ photoanodes were examined. The theoretical photocurrent densities (*J*_abs_), correlated with PEC activity and UV–Vis absorption, are 6.77, 6.61, and 6.56 mA cm^−2^ for Ru_0.51_-CoFe-LDH/BiVO_4_, CoFe-LDH/BiVO_4_, and BiVO_4_ photoanodes, respectively (Fig. S12). Photocurrent densities were also measured in KPi electrolyte with Na_2_SO_3_ as a hole scavenger to reduce surface charge recombination (Fig. S13). The charge separation efficiency (*η*_sep_) of Ru_0.51_-CoFe-LDH/BiVO_4_ photoanode increases to 87.4% at 1.23 V vs. RHE, which is higher than reported WCoFe-LDH/BiVO_4_ [36], demonstrating that the introduction of Ru atomic active sites enhances the interface charge separation (Fig. 5a). The charge injection efficiency of Ru_0.51_-CoFe-LDH/BiVO_4_ reach 76% at 1.23 V vs. RHE, outperforming the other two photoanodes (Fig. 5b). Open circuit potential (OCP) decay kinetics were measured to further assess the photoanodes. The OCP value is 0.15 V_RHE_ for Ru_0.51_-CoFe-LDH/BiVO_4_ photoanodes, higher than CoFe-LDH/BiVO_4_ (0.11 V_RHE_) and BiVO_4_ (0.08 V_RHE_) (Figs. 5c and S14), determined by the difference between the quasi-Fermi level under illumination and the electrolyte’s redox potential [40]. The higher OCP indicates that the incorporation of Ru sites reduces surface trap states between Ru_0.51_-CoFe-LDH and BiVO_4_, mitigating Fermi level pinning and enhancing the driving force for hole injection into the electrolyte. Moreover, the elevated OCP suggests an increased concentration of photogenerated carriers in Ru_0.51_-CoFe-LDH, consistent with the Mott-Schottky analysis. To clarify charge recombination at the semiconductor/electrolyte interface, carrier transient lifetimes were measured upon light removal. The Ru_0.51_-CoFe-LDH/BiVO_4_ photoanode displays the shortest transient lifetime (0.213 s), compared to CoFe-LDH/BiVO_4_ (0.297 s) and BiVO_4_ (0.376 s) (Fig. 5d), indicating that the Ru atomic active sites transfer charge completely in a shorter time than Co sites or Fe sites [41]. Transient photocurrent curves show an increase in the* i*/*i*₀ value from 0.714 for BiVO_4_ to 0.921 for CoFe-LDH/BiVO_4_ and 0.962 for Ru_0.51_-CoFe-LDH/BiVO_4_, further confirming that Ru sites accelerate carrier separation (Fig. 5e).

Steady-state photoluminescence (PL) spectra reveal that Ru_0.51_-CoFe-LDH/BiVO_4_ has the weakest fluorescence intensity (Fig. 5f), indicating effective suppression of electron–hole recombination. In Fig. S15, electrochemically effective surface areas (ECSA) were calculated from electrochemical double layer capacitance (*C*_dl_). CV curves at various scan rates shows *C*_dl_ values of 60.5 μF cm⁻^2^ for Ru_0.51_-CoFe-LDH/BiVO_4_, 48.5 μF cm⁻^2^ for CoFe-LDH/BiVO_4_, and 41 μF cm⁻^2^ for /BiVO_4_ (Fig. 5g), with the largest ECSA for Ru_0.51_-CoFe-LDH/BiVO_4_, due to its abundant Ru atomic sites. Polarization curves measured in the dark revealed the Tafel slope for evaluating OER kinetics. Ru_0.51_-CoFe-LDH/BiVO_4_ demonstrates lower onset potential and higher current density than CoFe-LDH/BiVO_4_ and BiVO_4_, indicating superior OER activity (Fig. 5h). Figure 5i exhibits that the Tafel slope is consistent with the EIS result, indicating a fast charge transfer process of Ru_0.51_-CoFe-LDH/BiVO_4_ photoanode in the water oxidation reaction [42].

**Fig. 6:**
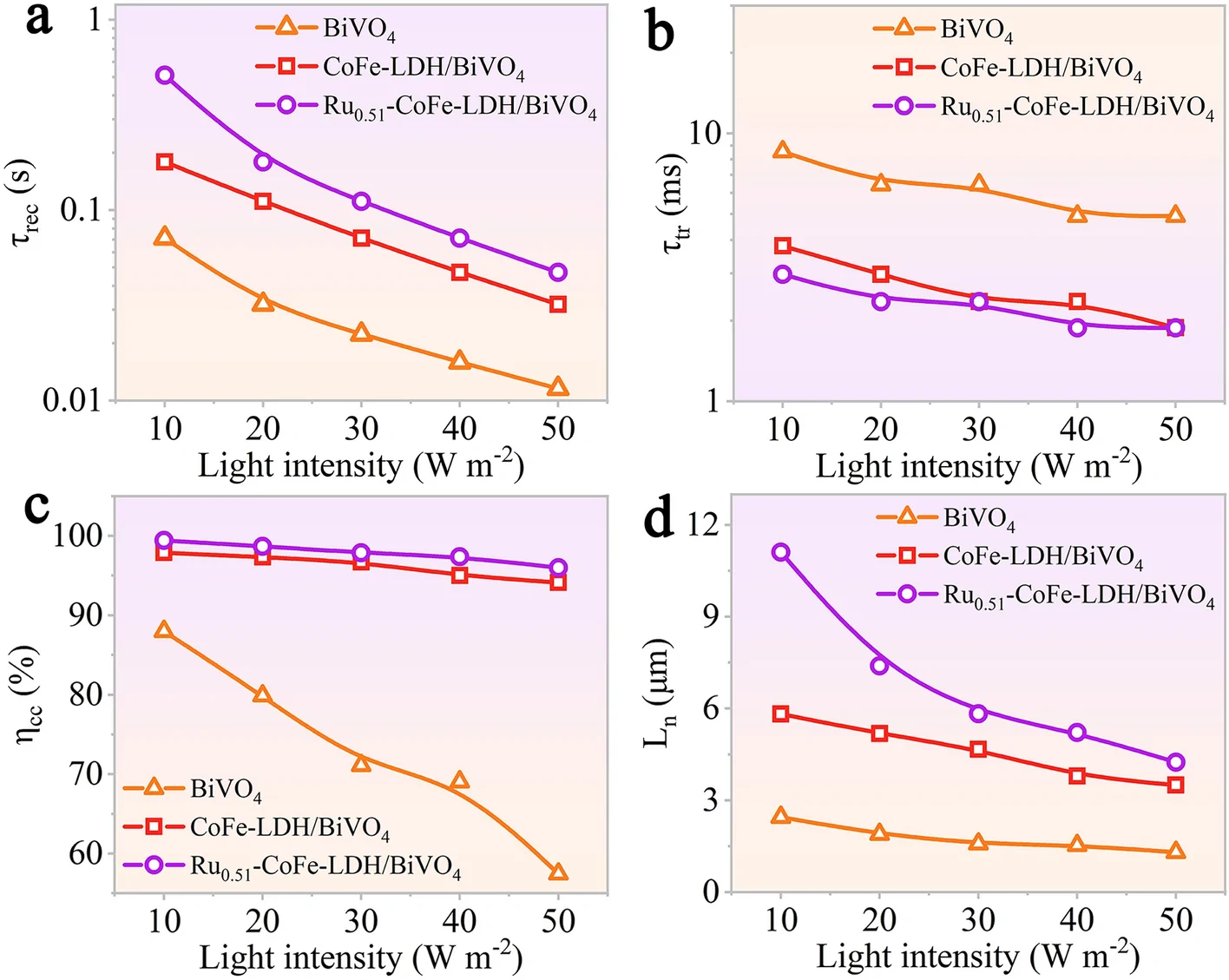
**a** Charge recombination time constant, **b** charge transfer time constant, **c** charge collection efficiency, **d** charge average diffusion distance of three photoanodes

To further explore carrier separation and transfer dynamics, controlled intensity modulated photovoltage and photocurrent spectroscopy (CIMVS/CIMPS) were employed to investigate the carrier separation ability and transfer dynamics. Fig. S16 shows the CIMVS and CIMPS Nyquist plots at different light intensities. The charge recombination (*τ*_rec_) and transfer (*τ*_tr_) time constants were calculated using the following equations [43]:

1 $$\tau_{{{\mathrm{rec}}}} = \, \frac{1}{{2\pi f_{{\min ,{\mathrm{CIMVS}}}} }}$$

2 $$\tau_{{{\mathrm{tr}}}} = \, \frac{1}{{2\pi f_{{\min ,{\mathrm{CIMPS}}}} }}$$

where *f*_min*,*CIMVS_ and *f*_min_*,*_CIMPS_ represent the frequency of the lowest point of the imaginary part feature (lower semicircle) of CIMVS and CIMPS curves, respectively. *τ*_rec_ represents carrier lifetime, while *τ*_tr_ indicates the time for electrons to transfer to the circuit. Figure 6a, b shows that both *τ*_rec_ and *τ*_tr_ decrease with light intensity, likely due to electrons transitioning from deep to shallow energy states, enhancing electron transport but increasing recombination [44]. Notably, the Ru_0.51_-CoFe-LDH/BiVO_4_ photoanode consistently exhibits the longest carrier lifetime and shortest electron transfer time across all light intensities compared to CoFe-LDH/BiVO_4_ and BiVO_4_ photoanode.

To further investigate the charge separation and transfer process, charge collection efficiency (*η*_cc_) and charge average diffusion distance (*L*_n_) were evaluated. These parameters were calculated using time constants, with the following equations:

3 $$\eta_{cc} = 1 - \frac{{\tau_{{{\mathrm{tr}}}} }}{{\tau_{{{\mathrm{rec}}}} }}$$

4 $${\mathrm{L}}_{{\mathrm{n}}} = \, d\sqrt {\frac{{\tau_{{{\mathrm{tr}}}} }}{{2.35\tau_{{{\mathrm{rec}}}} }}}$$

where *d* is the thickness of the photoanode film [44]. Figure 6c illustrates superior charge collection efficiency of Ru_0.51_-CoFe-LDH/BiVO_4_ (~ 100%) compared to CoFe-LDH/BiVO_4_ and bare BiVO_4_. It is noteworthy that the *L*_n_ of the three photoanodes surpassed their respective film thickness, implying a negligible impact of film thickness on the charge collection of the photoanodes (Figs. 6d and S2). These results further confirm that the Ru_0.51_-CoFe-LDH/BiVO_4_ photoanode displays more efficient photogenerated charge separation and transfer than CoFe-LDH/BiVO_4_ and bare BiVO_4_ photoanode.

### Theoretical Insights

**Fig. 7:**
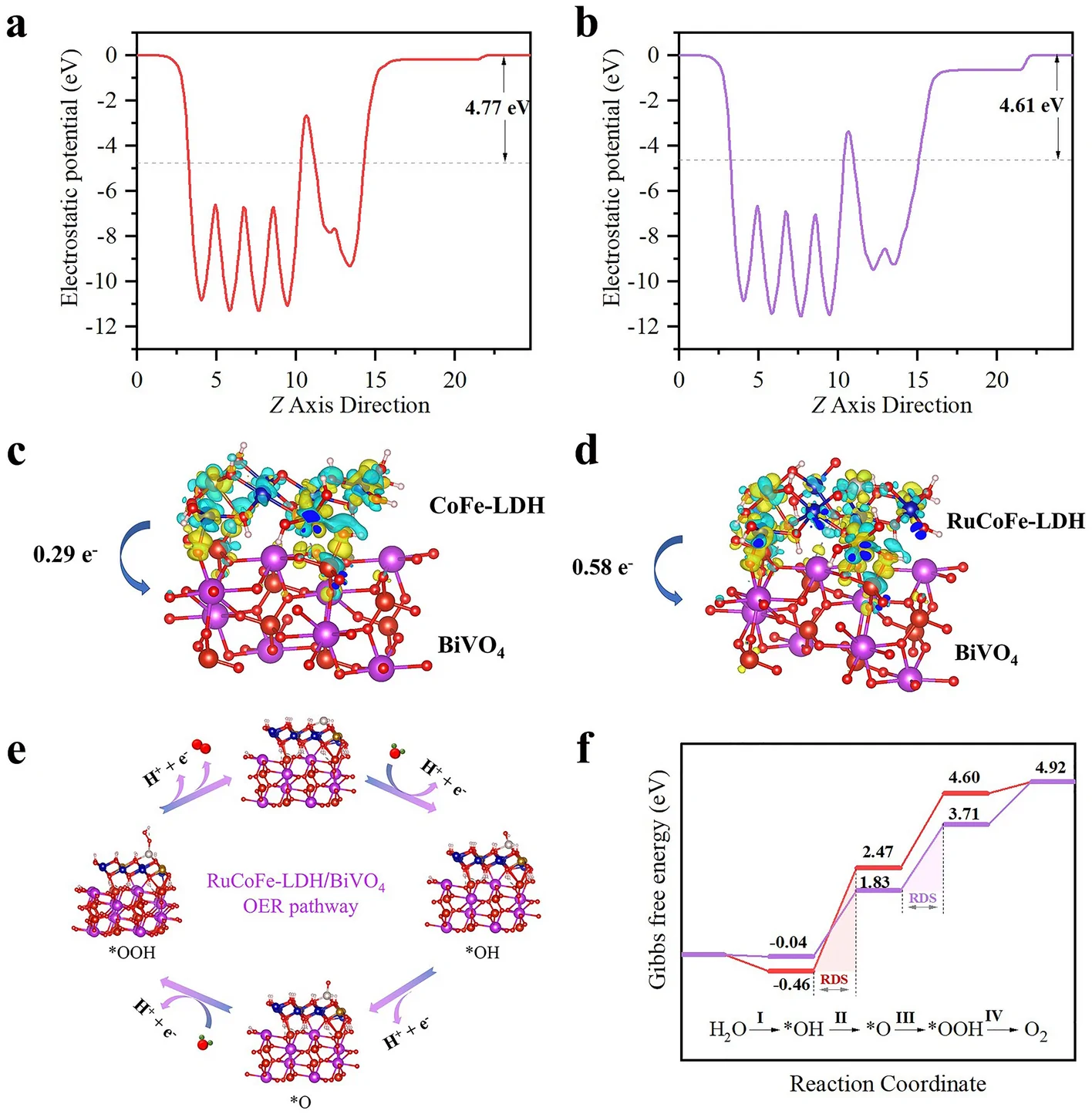
Electrostatic potential plot of **a** CoFe-LDH/BiVO_4_, and **b** RuCoFe-LDH/BiVO_4_. Differential charge density of **c** CoFe-LDH/BiVO_4_ and **d** RuCoFe-LDH/BiVO_4_. **e** Four electron step reaction pathway models with Ru sites as OER active sites; **f** OER Gibbs free energy diagrams of RuCoFe-LDH/BiVO_4_ and CoFe-LDH/BiVO_4_ photoanode films at 0 V

To gain a profound understanding of how the introduction of Ru single-atom active sites improved the OER performance of photoanode films, DFT + U calculations were conducted for the CoFe-LDH/BiVO_4_ and RuCoFe-LDH/BiVO_4_ heterojunction models (Fig. S17). The electrostatic potentials along the *Z*-axis were evaluated for the composite systems as well as for the individual BiVO_4_ and LDH components. As shown in Figs. 7a, b and S18, the work functions of CoFe-LDH and RuCoFe-LDH lie between those of the corresponding isolated LDH and BiVO_4_ structures, confirming the formation of heterojunction interface. Notably, RuCoFe-LDH exhibits a lower work function than CoFe-LDH, and similarly, RuCoFe-LDH/BiVO_4_ shows a lower work function than CoFe-LDH/BiVO_4_, in agreement with the Mott–Schottky results in Fig. 4f. Charge density difference analysis (Fig. 7c, d) further reveals that approximately 0.29 e⁻ is transferred from CoFe-LDH to BiVO_4_ in the CoFe-LDH/BiVO_4_ system, whereas the charge transfer increases to about 0.58 e⁻ in the RuCoFe-LDH/BiVO_4_ system. In addition, the density of states (DOS) plot of RuCoFe-LDH displays a smaller bandgap and a higher Fermi level compared to CoFe-LDH (Fig. S19), consistent with the trends observed in Fig. 3b, d. These results collectively indicate a stronger built-in electric field in RuCoFe-LDH/BiVO_4_ relative to CoFe-LDH/BiVO_4_. It is manifest that the accumulation and depletion of charge density predominantly transpire at Ru atomic loci.

The projected density of states (PDOS) analysis reveals that the orbital overlap between the Ru sites and reaction intermediates (*OH, *O, and *OOH) is higher compared to the Co and Fe sites, signifying a stronger adsorption capability of the intermediates at the Ru sites (Fig. S20). This implies that throughout the PEC water splitting process of the Ru_0.51_-CoFe-LDH/BiVO_4_ photoanode, the incorporation of single Ru atoms on the surface alters the charge distribution and establish an innovative charge transfer pathway, consequently facilitating enhanced charge separation. At the same time, the adsorption of reaction intermediates (*OH, *O and *OOH, * is the catalytic active site) is detected to simulate the OER process of the photoanode (Figs. 7e and S21). Free-energy calculations, performed using the VASPsol model with a dielectric constant of 78.4 demonstrate that the incorporation of Ru single atoms reduces the energy barrier for the *OH to *O transition in the RuCoFe-LDH/BiVO_4_ system (Figs. 7f and S22). The potential-determining step is identified as *O → *OOH, with a barrier of 1.88 eV. These findings confirm that single Ru atomic sites serve act as the primary active centers, effectively lowering energy barrier for OER and promoting the surface oxygen evolution reaction kinetics of the Ru_0.51_-CoFe-LDH/BiVO_4_ photoanode.

## Conclusion

In summary, we develop a novel strategy involving the integration of a single metal ruthenium coupled with a CoFe-LDH cocatalyst (Ru_0.51_-CoFe-LDH) onto a BiVO_4_ semiconductor substrate. AC-HADDF-STEM images and spectroscopic analysis unequivocally validate the monodisperse characteristics of ruthenium, demonstrating their presence as "Ru-OH" moieties on the LDH surface. Experimental results combined with DFT analysis reveal that the single Ru atoms intercalation in the CoFe-LDH results in significant charge redistribution for Ru_0.51_-CoFe-LDH to improve binding energy between the active sites and intermediates. Simultaneously, the upward shift in the band edge position of Ru_0.51_-CoFe-LDH induces a more pronounced band bending at the n–n junction formed with BiVO_4_ substrate, expediting the separation and transfer of photogenerated electron–hole pairs at the interface. Impressively, the Ru_0.51_-CoFe-LDH/BiVO_4_ photoanode achieves a high photocurrent density of 4.51 mA cm^−2^ at 1.23 V vs. RHE, coupled with a high charge injection efficiency (76%) and exceptional long-term operational stability (10 h) at an electrolyte containing 0.1 M NaVO_3_. This work leverages LDH as vehicle-assisted cocatalysts, offering a fresh perspective for the development of semiconductor photoanode devices with single atomic sites.

## Supplementary Information

Below is the link to the electronic supplementary material.

Supplementary file1 (DOCX 14105 KB)
