# Supplementary material for: Single-Atom Ru in CoFe-LDH Drives Efficient Charge Separation on BiVO4 for Solar Water Splitting
Source: Nanomicro Lett. 2026 Jan 19;18:212. doi: 10.1007/s40820-025-02062-y (PMC12816489; doi:10.1007/s40820-025-02062-y)
Supplement: Supplementary file 1 — Supplementary file1 (DOCX 14105 KB) [file 40820_2025_2062_MOESM1_ESM.docx]

Supporting Information for

**Single-Atom Ru in CoFe-LDH Drives Efficient Charge Separation on BiVO_4_ for** **Solar Water Splitting**

Wenhui Deng^1,#^, Gaoshuang He^1,#^, Haozhi Zhou^2,#^, Wenhao He^1^, Lei Gan^1^, Chenyu Zhang^1^, Keke Wang^4^, Xiaoqing Qiu^1^, Yang Liu^1,*^, Wenzhang Li^1,3,*^

^1^ School of Chemistry and Chemical Engineering, Central South University, Changsha 410083, People’s Republic of China

^2^ School of Physical Science and Technology, ShanghaiTech University, Shanghai 201203, People’s Republic of China

^3^ Hunan Provincial Key Laboratory of Chemical Power Sources, Central South University, Changsha 410083, People’s Republic of China

^4^ School of Chemistry and Chemical Engineering, Hunan University of Science and Technology, Xiangtan 411100, People’s Republic of China

^#^Wenhui Deng, Gaoshuang He, and Haozhi Zhou contributed equally to this work.

Corresponding authors. E-mail: [yangliu_csu@csu.edu.cn](mailto:yangliu_csu@csu.edu.cn) (Yang Liu); [liwenzhang@csu.edu.cn](mailto:liwenzhang@csu.edu.cn) (Wenzhang Li)

**S1 Experimental Section**

## S1.1 Characterization

The crystallinity and growth direction of the photoanode were determined by X-ray powder diffractometer (XRD, Rigaku D/max 2250 VB) using Cu K*α* was used as the radiation source (40 kV, 450 mA, *λ* = 0.15406 nm). The surface morphology and thickness of the photoanode films were characterized by scanning electron microscopy (SEM, JEOL/JSM-7610FPlus), transmission electron microscopy (TEM) and high-resolution transmission electron microscopy (HRTEM, FEI Tecnai G2F20). Aberration-corrected high-angle annular darkfield scanning transmission electron microscopy (AC-HAADF-STEM) images were taken on a Thermo scientific Themis Z (3.2) with a spherical aberration corrector working at 300 kV. The actual contents of ruthenium in co-catalysts were determined by inductively coupled plasma optical emission spectrometry (ICP-OES). Zeta potential was obtained with a particle size and Zeta potential analyzer (Zeta sizer Nano ZS, Malvern Instruments) at room temperature. The chemical states and the energy band structures of the films were investigated by X-ray photoelectron spectrometer (XPS) and valence band X-ray photoelectron spectrometer (VB-XPS, ThermoFisher ESCALAB250Xi) with Mono Al-Kα source. The optical properties of the samples were tested by UV-Vis diffuse reflectance spectrometer (UV-Vis DRS, TU-1901). Fourier transform infrared (FT-IR,) spectra were confirmed by PerkinElmer Spectrum 2. Raman spectroscopy (i-Raman® Plus, B&W Tek Inc.) was measured using a confocal Raman microscope with a 785 nm laser source.

## S1.2 Photoelectrochemical measurements

The standard three-electrode system was used to evaluate the photoelectrochemical performance of the sample at 25 °C by using the Zahner Zennium (Zahner, Germany) electrochemical workstation. The working electrode was the synthesized BiVO_4_ photoanode, the counter electrode was the platinum electrode, and the saturated silver/silver chloride (Ag/AgCl) was the reference electrode. The electrolyte was KPi solution (KH_2_PO_4_/K_2_HPO_4_ buffer solution, pH = 7) without special instructions. Sodium sulfite (Na_2_SO_3_) was used as a hole sacrificial agent (0.2 M Na_2_SO_3_ + 0.2 M KPi) to detect how many photogenerated holes can be transferred to the surface of the photoanode to react to obtain carrier separation efficiency (*η*_sep_) and injection efficiency (*η*_inj_). All the potentials relative to the Ag/AgCl electrode were converted into reversible hydrogen electrode potentials by the **formula S1**.

The linear sweep voltammetry (LSV) curves were measured in the range of 0.2-1.4 V vs. RHE at a scan rate of 20 mVs^-1^. The incident photon-to-current conversion efficiency (IPCE) values at each wavelength were obtained by the monochromatic light generated by the monochromator under the irradiation of a 150 W xenon lamp. Photoelectrochemical impedance spectroscopy (PEIS) was the Nyquist diagram measured at 1.23 V vs. RHE in the measurement range of 100 mHz~100 KHz. The Mott-Schottky (M-S) curves were measured in a dark condition in the potential range of 0.1-0.5 V vs. RHE at a frequency of 1 KHz. The stability of the samples was evaluated in a closed reaction cell containing 0.5 M KBi (KBO_3_ buffer solution, pH = 9.2) with or without 0.1 M NaVO_3_ electrolyte at 1.23 V vs. RHE, and the cumulative generation of hydrogen and oxygen was monitored. The gas content in the reaction cell was detected by gas chromatograph (GC 5890N) every 20 minutes. The open circuit potential (OCP) curves measured the potential of the samples with or without AM 1.5 G irradiation in the open circuit state. The transient photocurrent curves of three photoanodes were measured under 1.23 V vs. RHE with or without illumination. Using LED (WLL01) lamp as the light source, the Zahner CIMP-2 system (Zahner XPOT) measured the controlled intensity modulated photocurrent spectrum (CIMPS) under different light intensities (10~50 W m^-2^, step size of 10 W m^-2^) at 1.23 V vs. RHE potential at 100 mHz~1 kHz frequency. The controlled Intensity modulated photovoltage spectroscopy (CIMVS) test method was similar to CIMPS, except that the bias voltage was not applied to the working electrode. It was worth noting that the AC light frequency needs to be set to ~10% of the direct stream light. The Cyclic voltametric (CV) curves were measured at different scan rates (20-100 mV s^−1^, step size of 20 mV s^−1^) in the potential range of 0.56-0.86 V vs. RHE under dark conditions. The polarization curves were the LSV curve measured under dark conditions.

## S1.3 Computational details

All DFT computations were performed by the Vienna A_b_-Initio Simulation Package (VASP). The projector-augmented wave pseudopotentials method was used to describe electron-ion interactions. The Perdew-Burke Ernzerh (PBE) exchange correlation, which incorporates the on-site Coulomb Repulsion U term, was utilized. In the current study, the value of U is 4.3 for Fe, 4.0 for Co, and 2.4 for Ru. The U values are chosen based on existing literature [S1, S2]. BiVO_4_ slab was built utilizing the (110) slab crystal plane based on the TEM result. CoFe-LDH slabs were built utilizing the (001) crystal plane, and Ru-CoFe-LDH slabs were created by loading the ruthenium hydroxyl complex onto the (001) crystal plane of CoFe-LDHs based on the EXAFS results. The heterojunction models were constructed with consideration of both oxygen and metal terminations on the CoFe-LDH slab for the calculations. CoFe-LDH slabs were built utilizing the (001) crystal plane, and Ru-CoFe-LDH slabs were created by loading the ruthenium hydroxyl complex onto the (001) crystal plane of CoFe-LDHs. To further understand the activity, both O and metal terminations were considered for the CoFe-LDH slab. A spin-polarized method was used. A vacuum of 15 Å was applied along the z-axis. To optimize the structure, the energy change threshold was set to 10^−5^ eV, the atoms were relaxed until the force acting on each atom was less than 0.02 eV Å^−1^, the plane wave cutoff was set to 400 eV, and the van der Waals (vdW) correction was used in the modeling. The weak interaction was represented using the DFT+D3 approach and empirical correction based on Grimme's scheme [S3, S4]. For OER free energy calculation, VASPsol was used with EB_K=78.4 [S5].

Usually, the OER process usually divided into four reaction steps:

* + H_2_O → *OH + H^+^ + e^-^ ∆*G_1_*

*OH → *O + H+ + e^-^ ∆*G_2_*

*O + H_2_O → *OOH + H^+^ + e^-^ ∆*G_3_*

*OOH → O_2_ + H^+^ + e^-^ + * ∆*G_4_*

Here, * represents a surface-active site.

∆*G_1_* = ∆*G_*OH_*

∆*G_2_* = ∆*G_*O_* - ∆*G_*OH_*

∆*G_3_* = ∆*G_*OOH_* - ∆*G_*O_*

∆*G_4_* = 4.92 - ∆*G_*OOH_*

# S2 ICP measurement

Dissolve 20 mg Ru_x_-CoFe-LDH with nitric acid and dilute it with deionized water into 50 mL clear and transparent solution, and take 2 mL to determine by inductively coupled plasma optical emission spectrometry. The relevant results are shown in **Table S1**.

**Table S1** ICP results and Ru atomic ratio of the Ru_x_-CoFe-LDH

| Samples | Ru concentration measured by ICP | Mass percentage of Ru |
| --- | --- | --- |
| Ru_2_-CoFe-LDH | 0.61 mg mL^-1^ | 0.15 wt.% |
| Ru_5_-CoFe-LDH | 2.06 mg mL^-1^ | 0.51 wt.% |
| Ru_20_-CoFe-LDH | 6.10 mg mL^-1^ | 1.52 wt.% |

# S3 Relevant calculation formulas

All reversible hydrogen potentials (relative to RHE) were converted from Ag/AgCl potentials according to the Nernst equation:

$E_{\text{RHE}}=E_{\text{Ag/AgCl}}+0.059\times\text{pH}+0.197$ (S1)

Where *E*_RHE_ referred to reversible hydrogen potential. *E*_Ag/AgCl_ was obtained potential versus Ag/AgCl.

The empirical formula of vanadium-oxygen (V-O) bond length and Raman stretching frequency was as follows[6]:

$v(\text{cm}^{-1})=21349\times e^{-1.9176R(\text{Å)}}$ (S2)

Where *v* was the Raman shift (cm^-1^) and R was the V-O bond length (Å).

BiVO_4_ was a direct bandgap optical transition semiconductor. Therefore, the Tauc plot can be obtained according to formula (S2) and the bandgap (*E*_g_) of BiVO_4_, CoFe-LDH/BiVO_4_ and Ru_0.51_-CoFe-LDH/BiVO_4_ photoanodes were determined to be 2.54eV. The relationship between conduction band and valence band of semiconductor was shown by formula (S3).

$(\alpha hv)^{2}=A(hv-E_{\text{g}})$ (S3)

$E_{\text{CB}}=E_{\text{VB}}+E_{\text{g}}$ (S4)

Where *α* was the UV-visible absorption coefficient of semiconductor materials, *h* was Planck's constant, *v* was the optical frequency, *A* was the constant coefficient, *E*_CB_ was the conduction band position, *E*_CB_ was the valence band position.

The applied bias photon to current efficiency (ABPE) was used to characterize the conversion efficiency of the photoelectrode, which can be calculated from the LSV curve under AM 1.5G sunlight. The formula was as follows:

$\text{ABPE (\%) = }\frac{\text{[(1.23 - }V_{\text{bias}}\text{)(V)}\times|J_{\text{light}}-J_{\text{dark}}\text{|(mA cm}^{\text{-2}}\text{)]}}{\text{P}_{\text{light}}(\text{mW cm}^{\text{-2}})}\times100\%$ (S5)

Among them, *V*_bias_ represented the external bias applied on the electrode, *J*_light_ and *J*_dark_ represented the photocurrent density and dark current density at the corresponding potential, *P*_light_ was the power density of AM 1.5G (100 mW cm^-2^).

The incident photon-to-current conversion efficiency (IPCE) represented the ratio of the number of electrons in the external circuit to the number of incident photons per unit time, which can be calculated by the following formula:

$\text{IPCE}(\%)=\frac{(J_{\text{light}}-J_{\text{dark}}\text{)(mA cm}^{\text{-2}}\text{)}\times1239.8(V\times\text{nm})}{\lambda(\text{nm})\times\text{P}_{\text{light}}(\text{mW cm}^{\text{-2}})}\times100\%$ (S6)

Where 1239.8 was obtained by dividing the product of *h* (Planck's constant, 6.626 × 10^-34^) and *c* (the speed of light, 3 × 10^8^) by the charge per unit electron (1.6 × 10^-19^), *λ* being the wavelength of the incident light.

At the same time, the photocurrent density can be estimated by integrating the IPCE value with the standard solar spectrum (ASTMG-173-03). The specific formula was as follows:

$J_{C}=\int\frac{\lambda\times IPCE(\lambda)\times E(\lambda)}{1240}d\lambda$ (S7)

Where *E*(λ) represented the light power density (mW cm^-2^) at wavelength λ.

Some equations for evaluating the photocurrent density were shown below:

$J_{\text{photo}}=J_{abs}\times\eta_{sep}\times\eta_{inj}$ (S8)

 (S9)

$\eta_{sep}=J_{\text{Na}_{2}\text{SO}_{3}}/J_{abs}$ (S10)

$\eta_{inj}=J_{\text{KPi}}/J_{\text{Na}_{2}\text{SO}_{3}}$ (S11)

$\eta_{LHE}=1-10^{-A(\lambda)}$ (S12)

Among them, *J*_photo_ represented the actual photocurrent density generated on the semiconductor electrode, *J*_abs_ was the photocurrent density when the absorbed photons are completely converted into current, *η*_sep_ (charge separation efficiency) represented the ratio of the photogenerated carriers that do not undergo bulk recombination inside the semiconductor to the photogenerated carriers generated during the entire photoexcitation process, *η*_inj_ (charge injection efficiency) was defined as the ratio of the photogenerated carriers transferred to the semiconductor/electrolyte interface to the photogenerated carriers that do not undergo bulk recombination inside the semiconductor. *q* was the charge of the electron, *h* was the Planck's constant, *c* was the speed of light, *Φ*_λ_ was the photon flux of AM 1.5G solar spectrum, *η*_LHE_ was the light absorption efficiency of monochromatic light. J_Na2SO3_ was the photocurrent density measured in 0.2 M KPi containing 0.2 M Na_2_SO_3_ electrolyte, which served as hole scavengers and ensures the hole injection rate approaching 100%. *J*_KPi_ was the photocurrent density measured in 0.2 M KPi electrolyte. The light absorbance coefficient constant (A) was related to UV-Vis spectroscopy.

The photogenerated carrier transfer lifetime (*τ*_n_) can be calculated by the following formula:

$\tau_{n}=-\frac{k_{B}T}{e}\left(\frac{d\text{OCP}}{d\text{t}}\right)^{-1}$ (S13)

Where *k*_B_, *T*, *e*, and dOCP/dt were derivatives of Boltzmann constant, temperature, one-electron charge, and OCP transient decay, respectively.

**S4 Supplementary Figures and Tables**


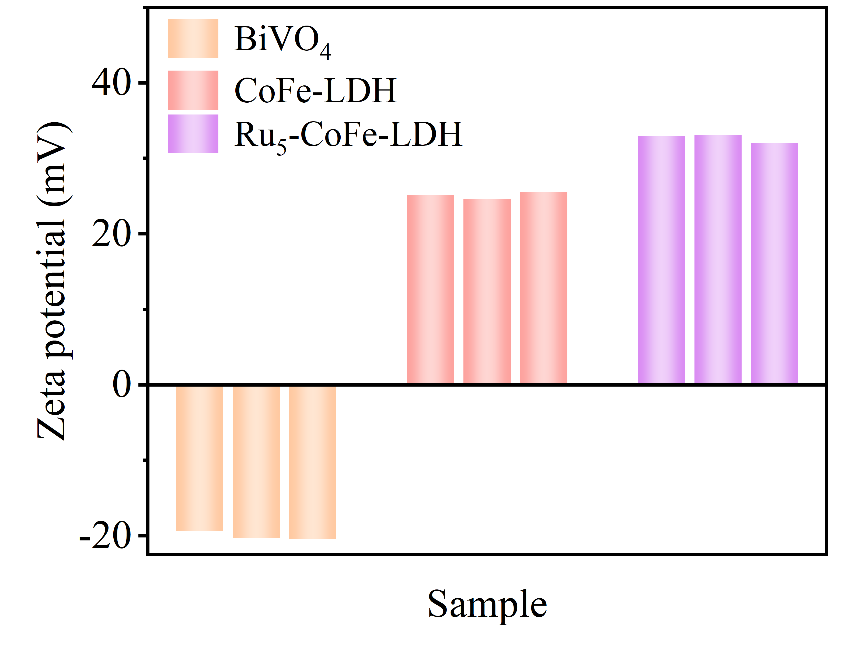


**Fig. S1** Zeta potential values of BiVO_4_ photoanode, CoFe-LDH and Ru_0.51_-CoFe-LDH nanosheets


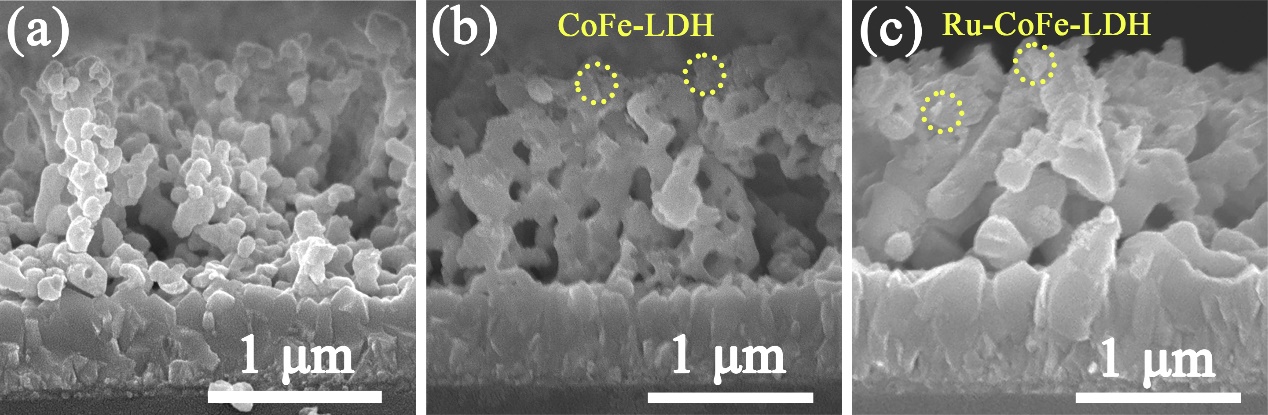


**Fig. S2** Cross-sectional SEM images of (**a**) BiVO_4_, (**b**) CoFe-LDH/BiVO_4_ and (**c**) Ru_0.51_-CoFe-LDH/BiVO_4_ films


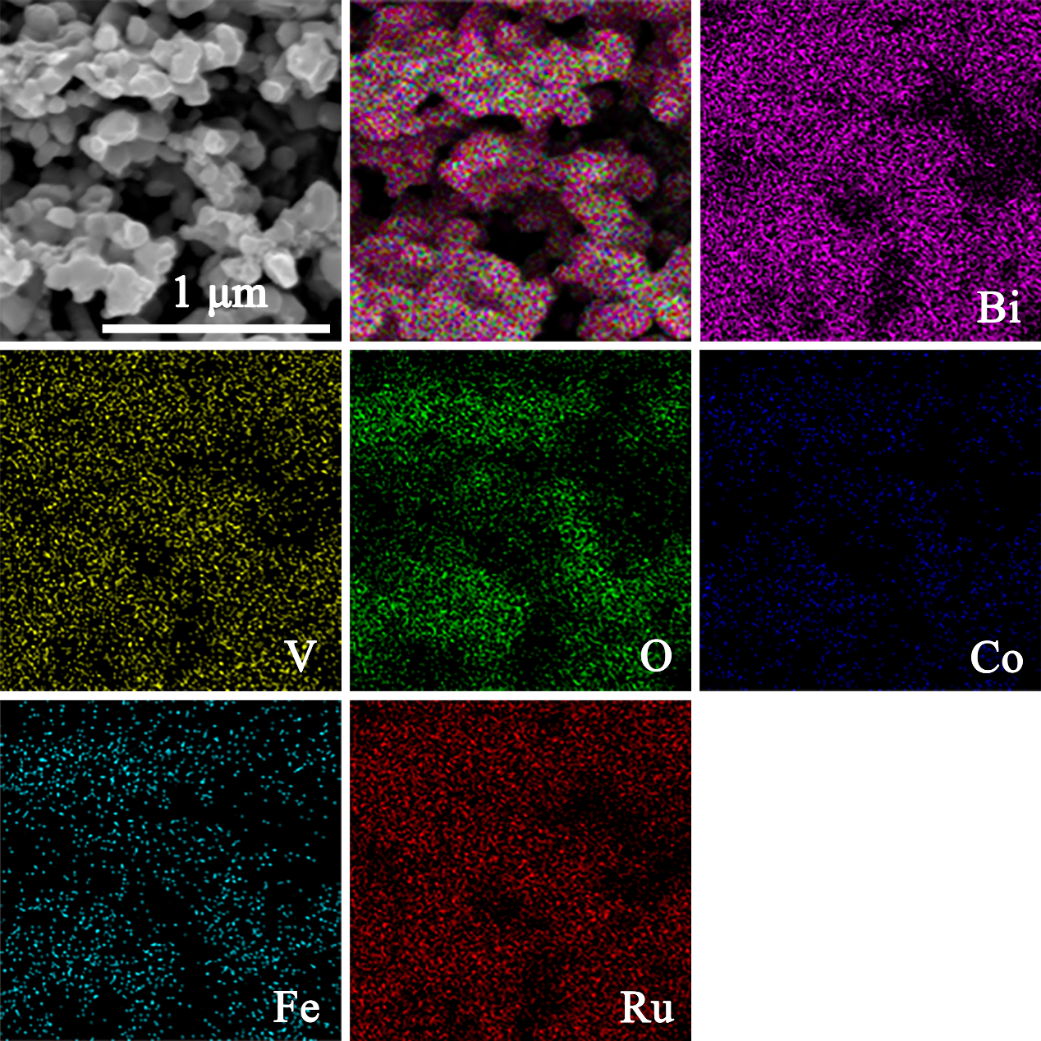


**Fig. S3** The corresponding EDS mapping images of Ru_0.51_-CoFe-LDH/BiVO_4_ films


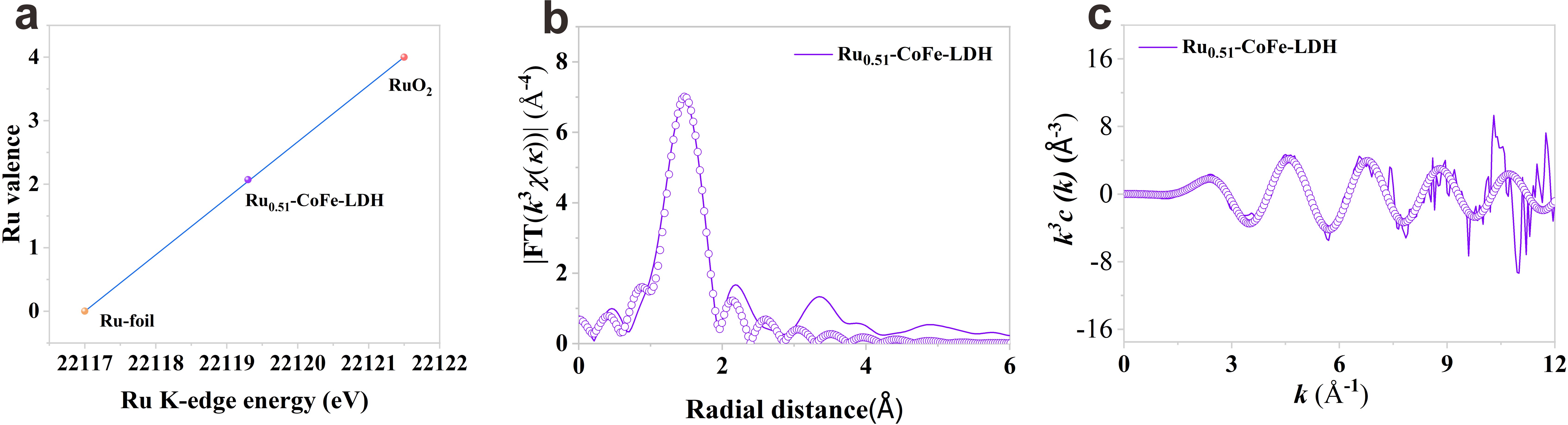


**Fig.** **S4** Linear fitting curve (**a**), FT-EXAFS fitting plots (**b**), and the EXAFS ꭓ(k) at Ru K-edge for Ru_0.51_-CoFe-LDH (**c**)


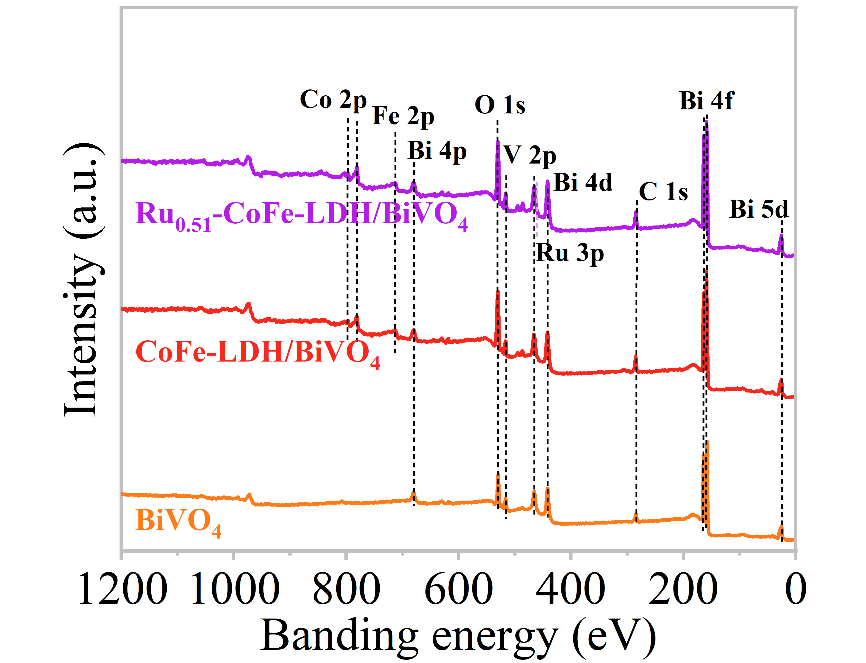


**Fig. S5** Total XPS survey spectra of BiVO_4_, CoFe-LDH/BiVO_4_ and Ru_0.51_-CoFe-LDH/BiVO_4_ films


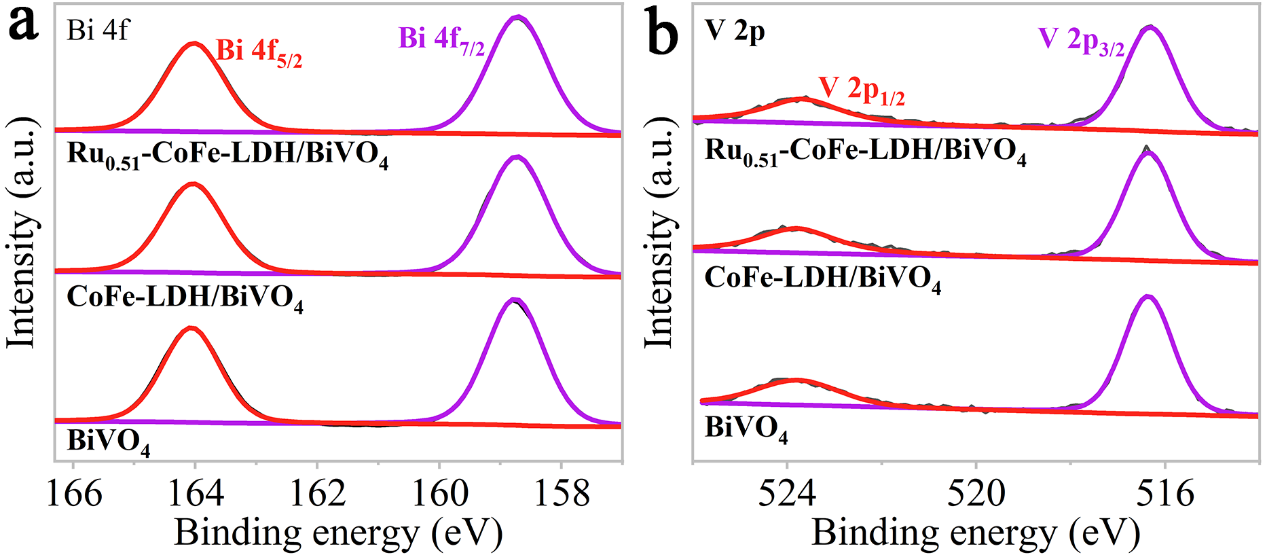


**Fig. S6** XPS spectra of Bi 4f and V 2p in three photoanodes


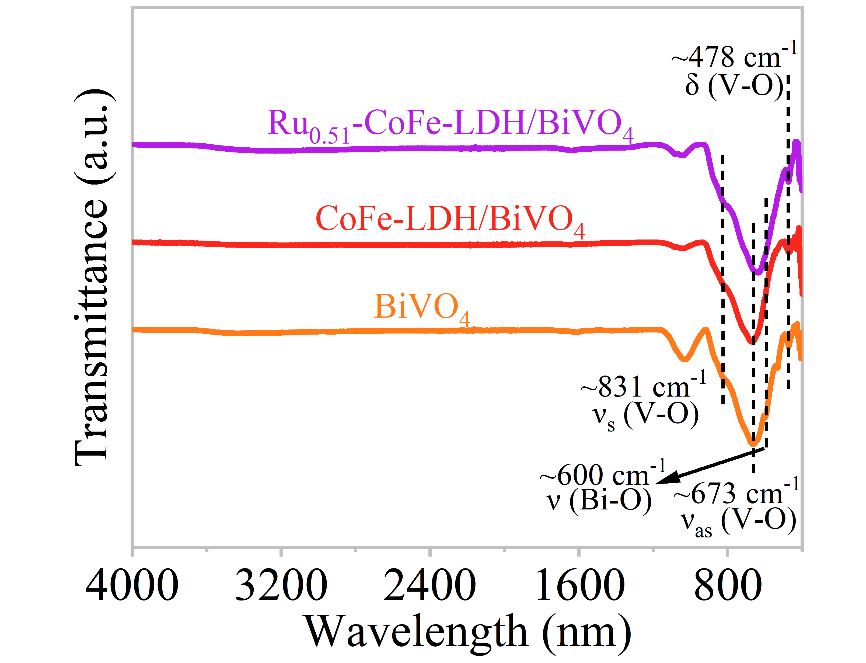


**Fig. S7** FT-IR absorption spectra of three photoanodes

Among them, the absorption peaks of ~831 cm^−1^ and ~673 cm^−1^ were mainly attributed to the VO_4_^3−^ unit ν_s_ (V-O) symmetric and ν_as_ (V-O) asymmetric stretching vibration mode [S7]. The peak signal near 600 cm^−1^ was mainly attributed to ν (Bi-O). More in, a weak signal observed in the absorption bands at ~478 cm^−1^ can be attributed to the δ (V-O) bending vibration of the VO_4_^3−^ unit [S8]. The above results prove that the synthesized BiVO_4_ belongs to the monoclinic scheelite phase.


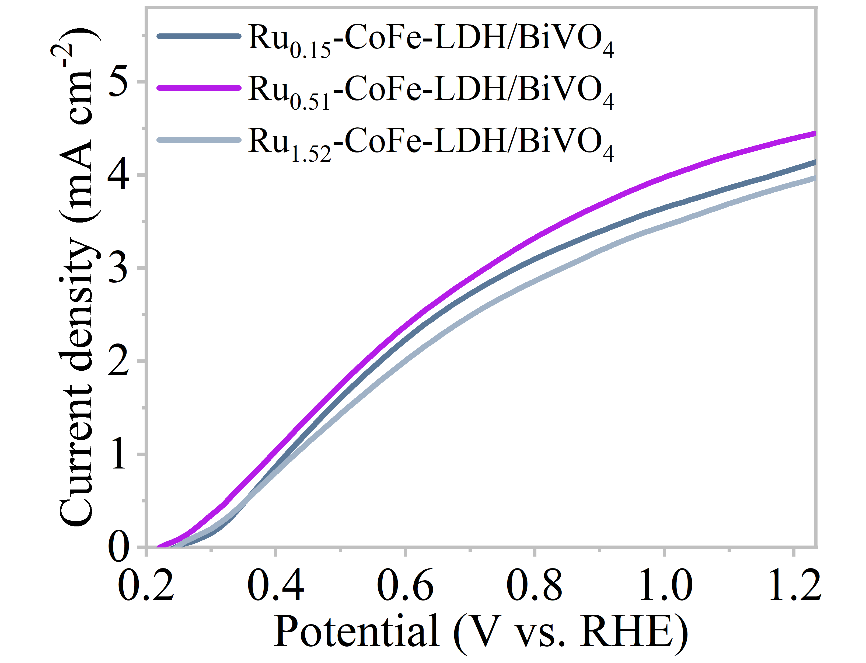


**Fig. S8** Linear sweep voltammetry curves of Ru_0.15_-CoFe-LDH/BiVO_4_, Ru_0.51_-CoFe-LDH/BiVO_4_ and Ru_1.52_-CoFe-LDH/BiVO_4_ photoanodes in 0.2 M KPi buffer solution


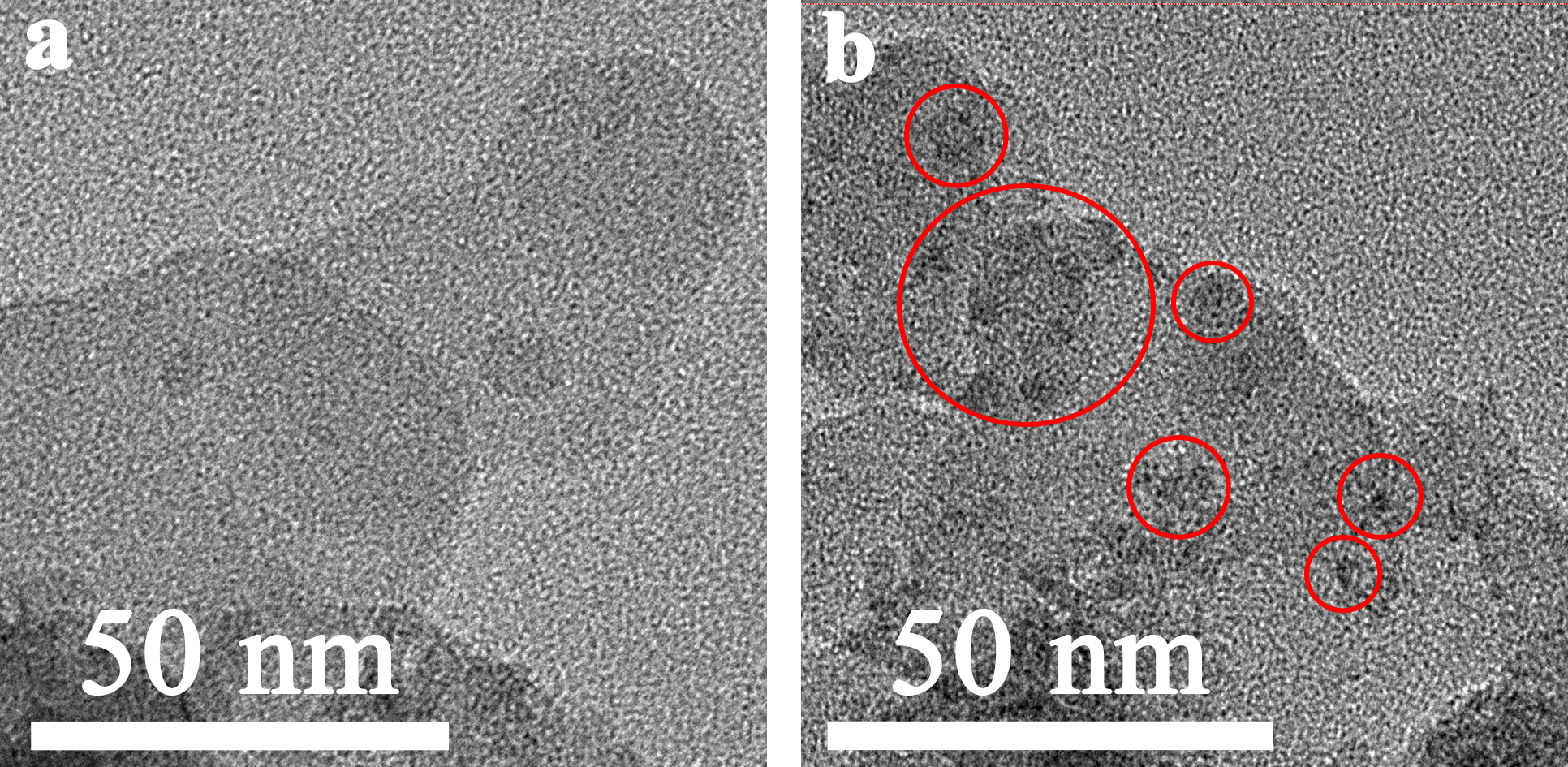


**Fig.** **S9** TEM images of (**a**) Ru_0.51_-CoFe-LDH and (**b**) Ru_1.52_-CoFe-LDH


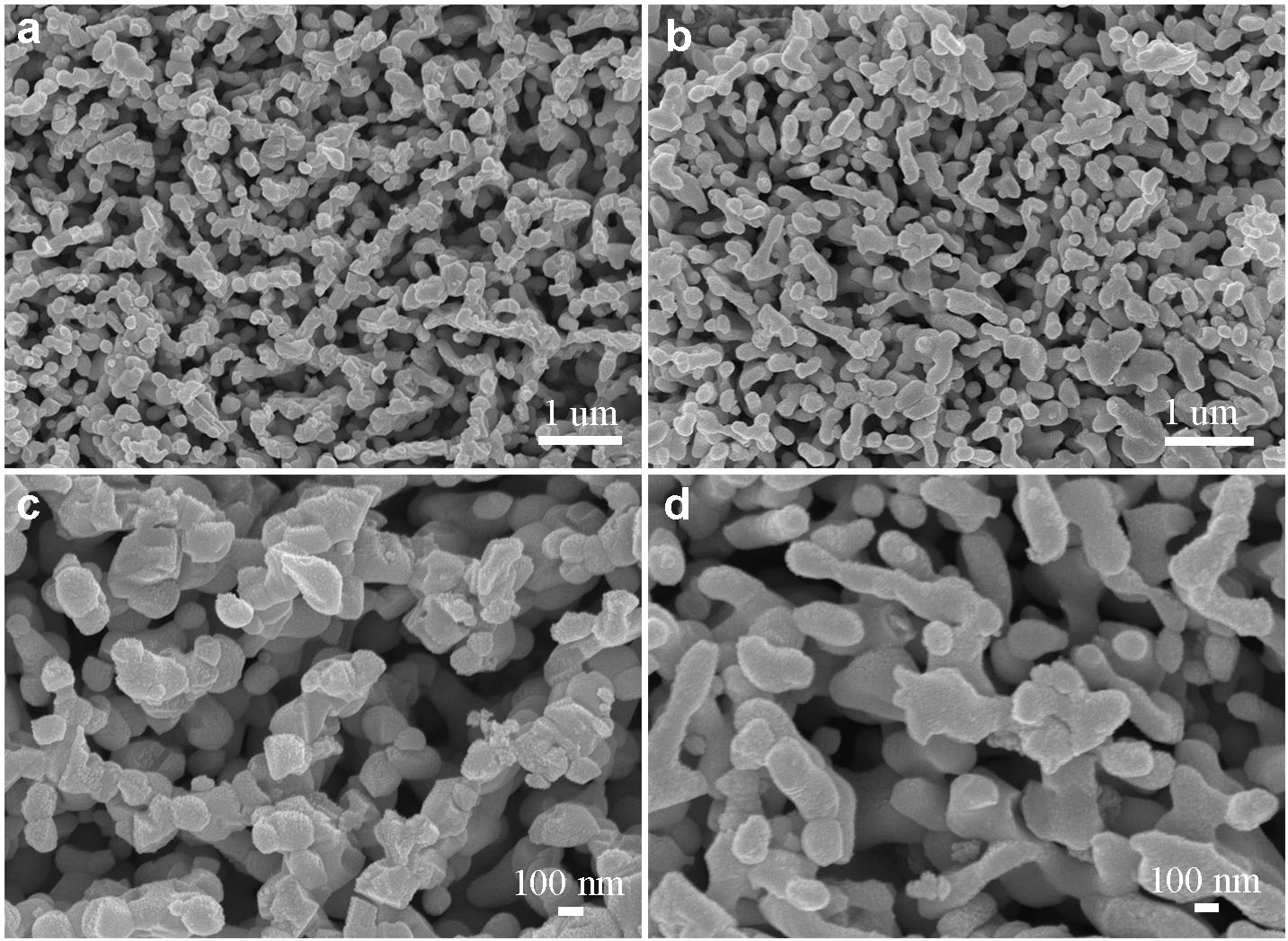


**Fig.** **S10** SEM images of Ru_0.51_-CoFe-LDH about (**a, c**) before i-t testing. (**b, d**) after i-t testing


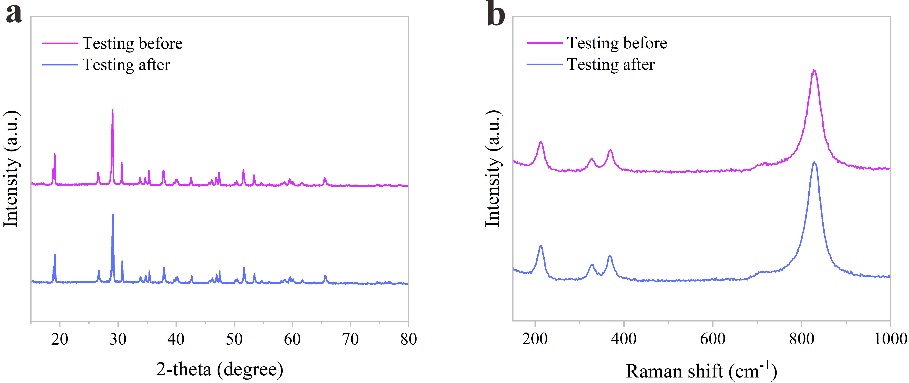


**Fig. S11** (**a**) XRD images of before and post-reaction of Ru_0.51_-CoFe-LDH. (**b**) Raman spectra of before and post-reaction of Ru_0.51_-CoFe-LDH


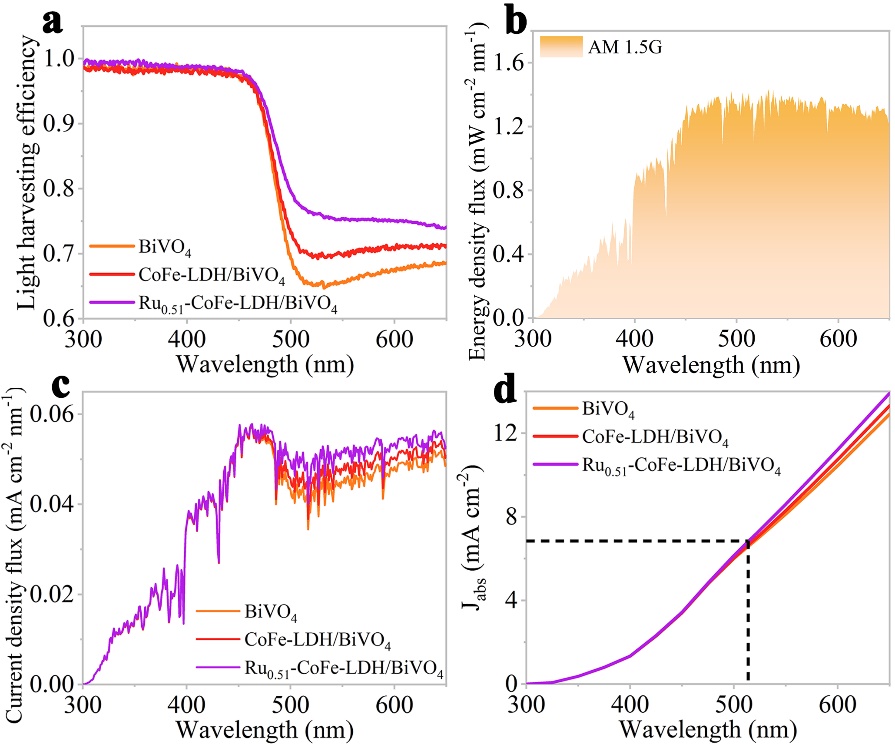


**Fig. S12** (**a**) Light harvesting efficiency spectra of BiVO_4_, CoFe-LDH/BiVO_4_ and Ru_0.51_-CoFe-LDH/BiVO_4_ photoanodes; (**b**) energy density flux of AM 1.5G standard solar spectrum; (**c**) calculated current density flux and (**d**) theoretical photocurrent density (*J*_abs_) of BiVO_4_, CoFe-LDH/BiVO_4_ and Ru_0.51_-CoFe-LDH/BiVO_4_ photoanodes


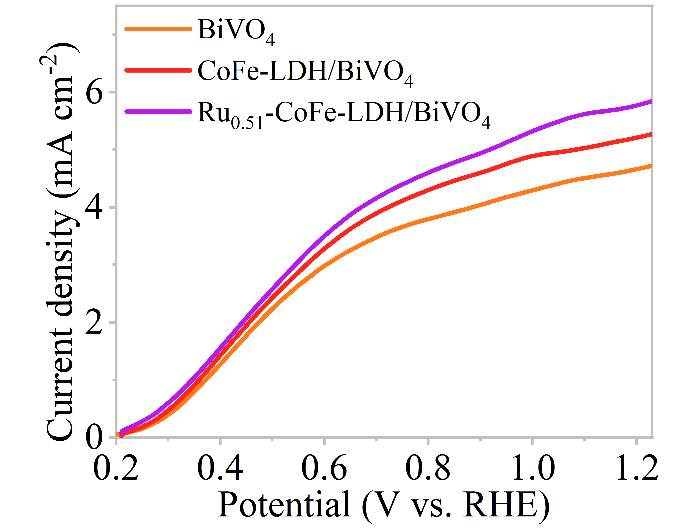


**Fig. S13** Linear sweep voltammetry curves of BiVO_4_, CoFe-LDH/BiVO_4_ and Ru_0.51_-CoFe-LDH/BiVO_4_ photoanodes in 0.2 M KPi buffer solution with 0.2 M Na_2_SO_3_


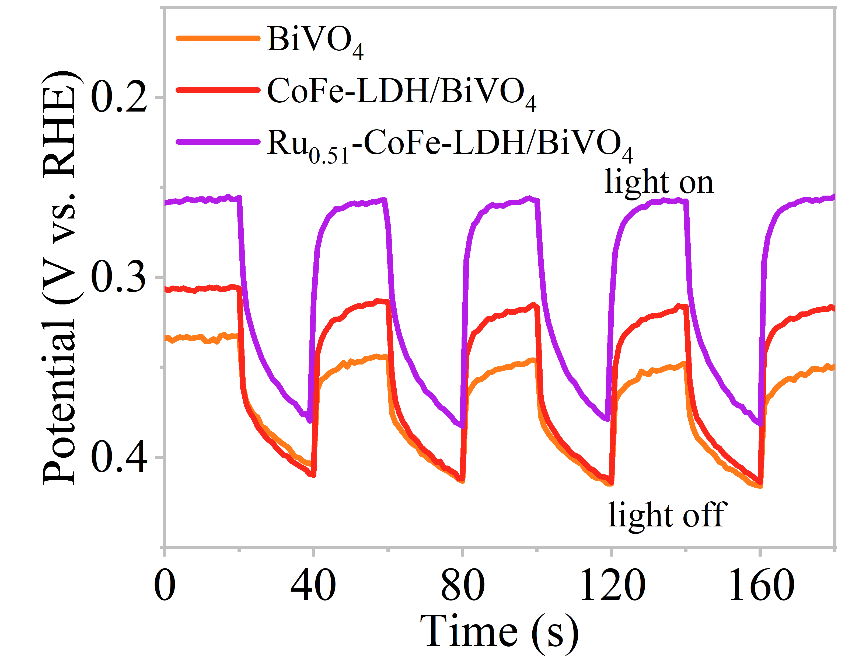


**Fig. S14** OCP transient decay curves under AM 1.5G illumination and in dark


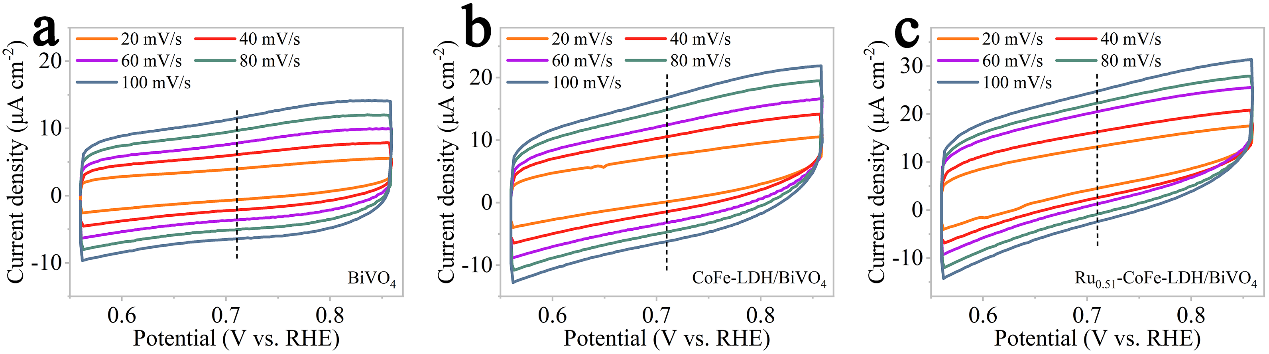


**Fig. S15** (**a**)-(**c**) CV curves at different scan


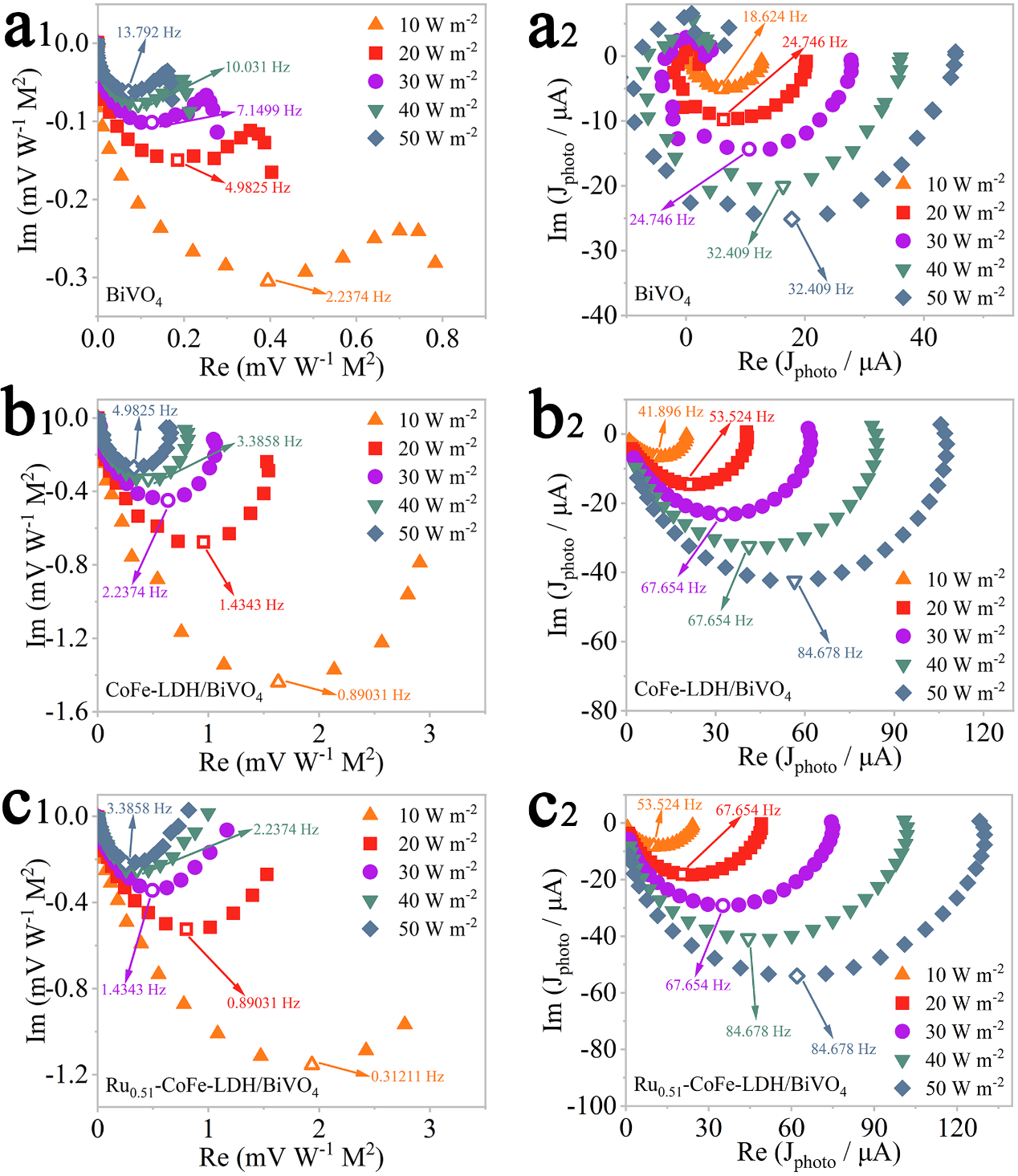


**Fig. S16** CIMVS spectra in Nyquist coordinates of (a_1_) BiVO_4_, (b_1_) CoFe-LDH/BiVO_4_ and (c_1_) Ru_0.51_-CoFe-LDH/BiVO_4_ films at different light intensities. CIMPS spectra in Nyquist coordinates of (a_2_) BiVO_4_, (b_2_) CoFe-LDH/BiVO_4_ and (c_2_) Ru_0.51_-CoFe-LDH/BiVO_4_ films at different light intensities


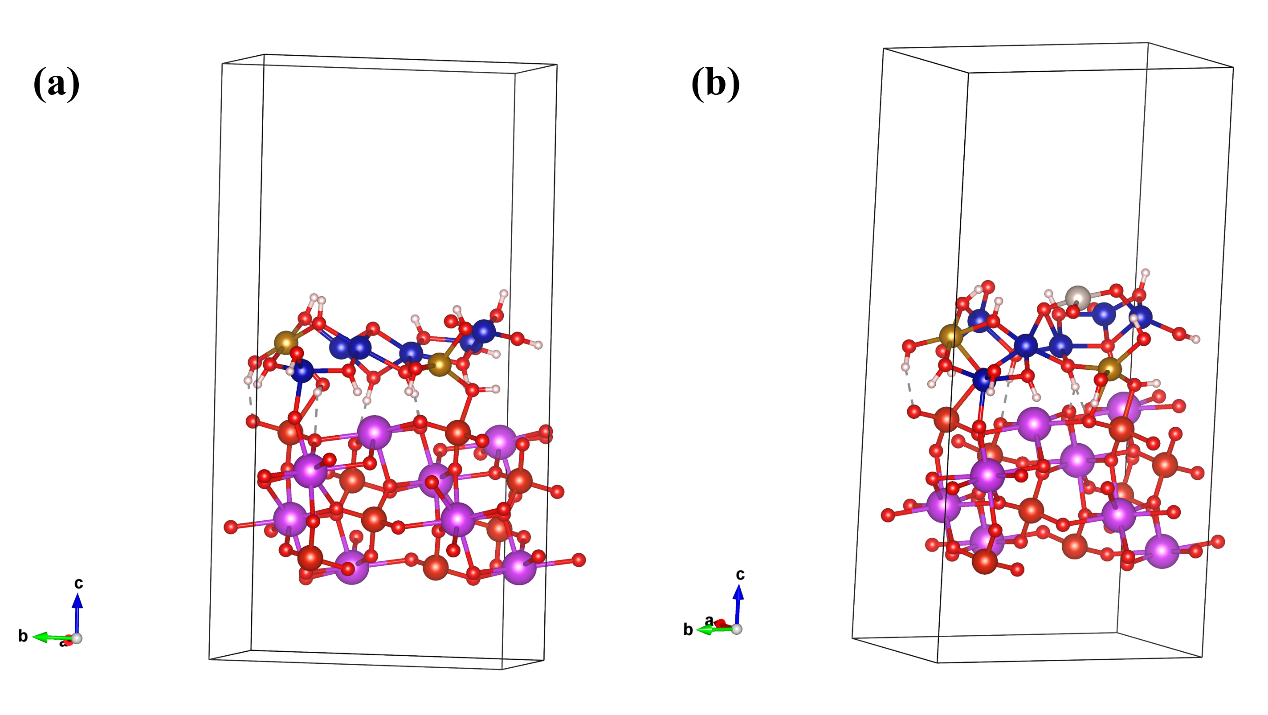


**Fig. S17** Constucted models of (**a**) CoFe-LDH/BiVO_4_ and (**b**) RuCoFe-LDH/BiVO_4_


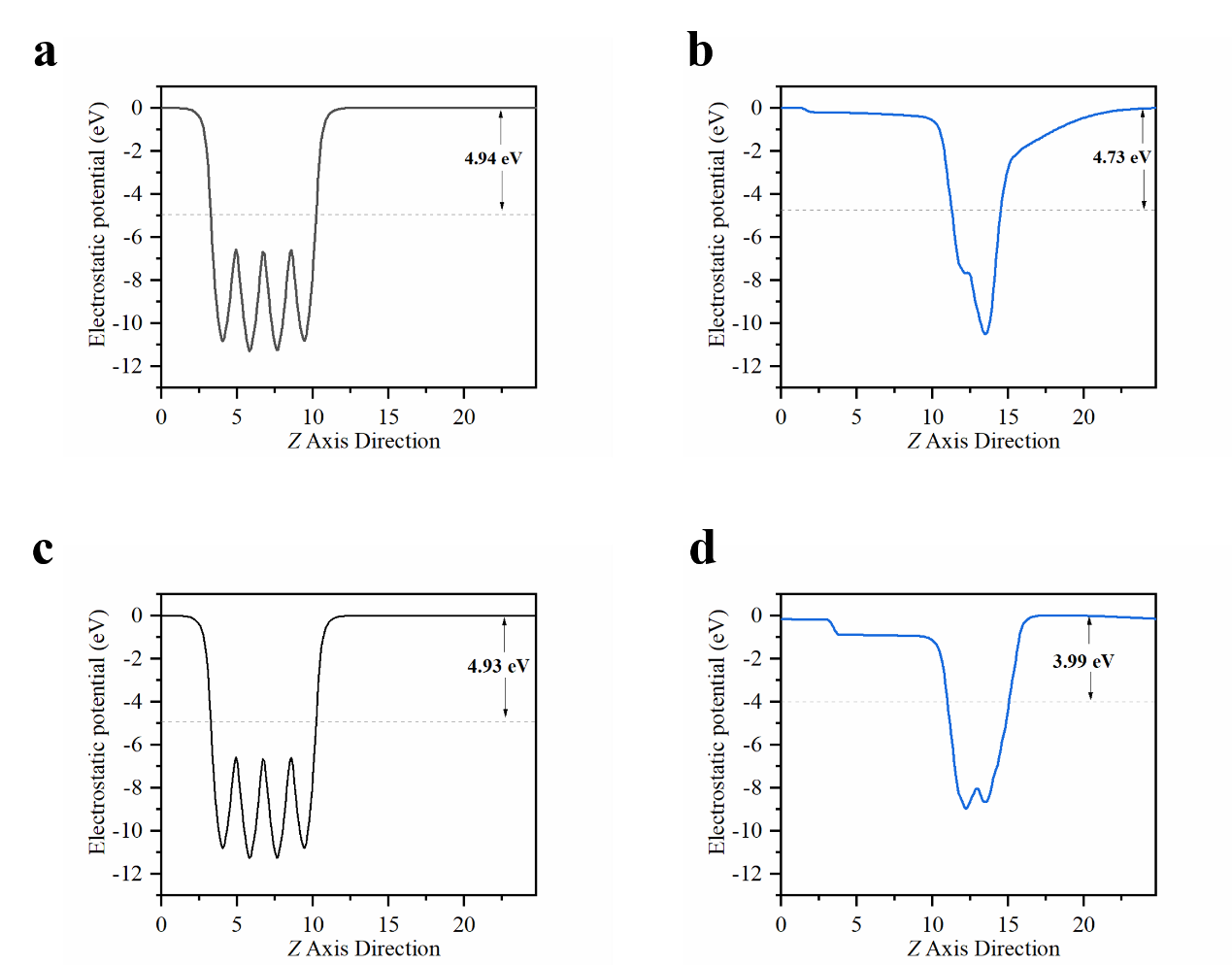


**Fig. S18** Electrostatic potential plot of (**a**) BiVO_4_ of CoFe-LDH/BiVO_4_, (**b**) CoFe-LDH, (**c**) BiVO_4_ of RuCoFe-LDH/BiVO_4_, and (**d**) RuCoFe-LDH


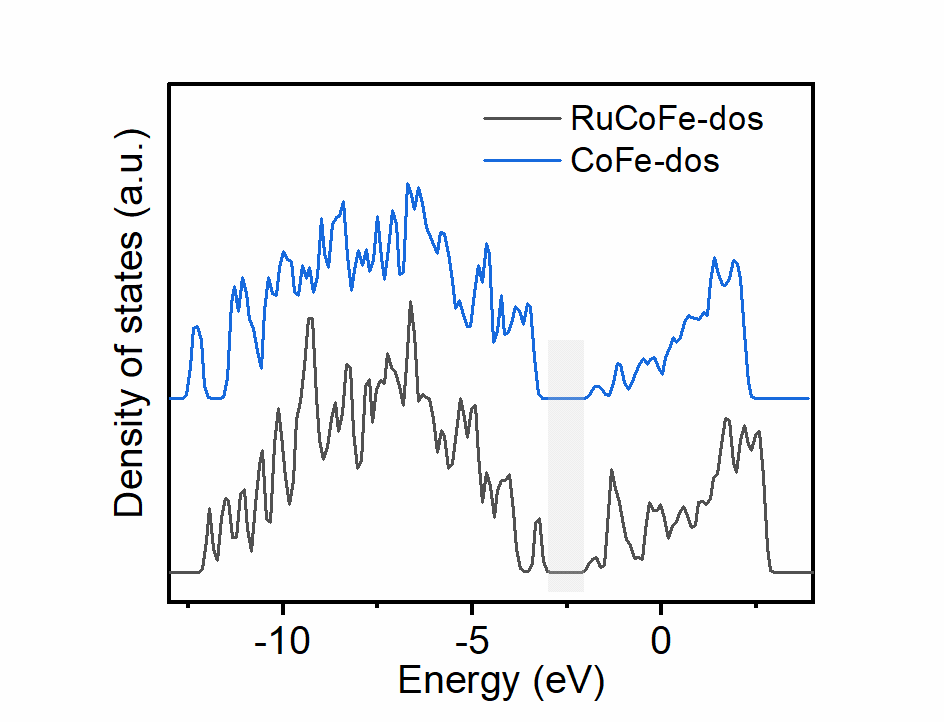


**Fig. S19** DOS plots of CoFe-LDH and RuCoFe-LDH

**
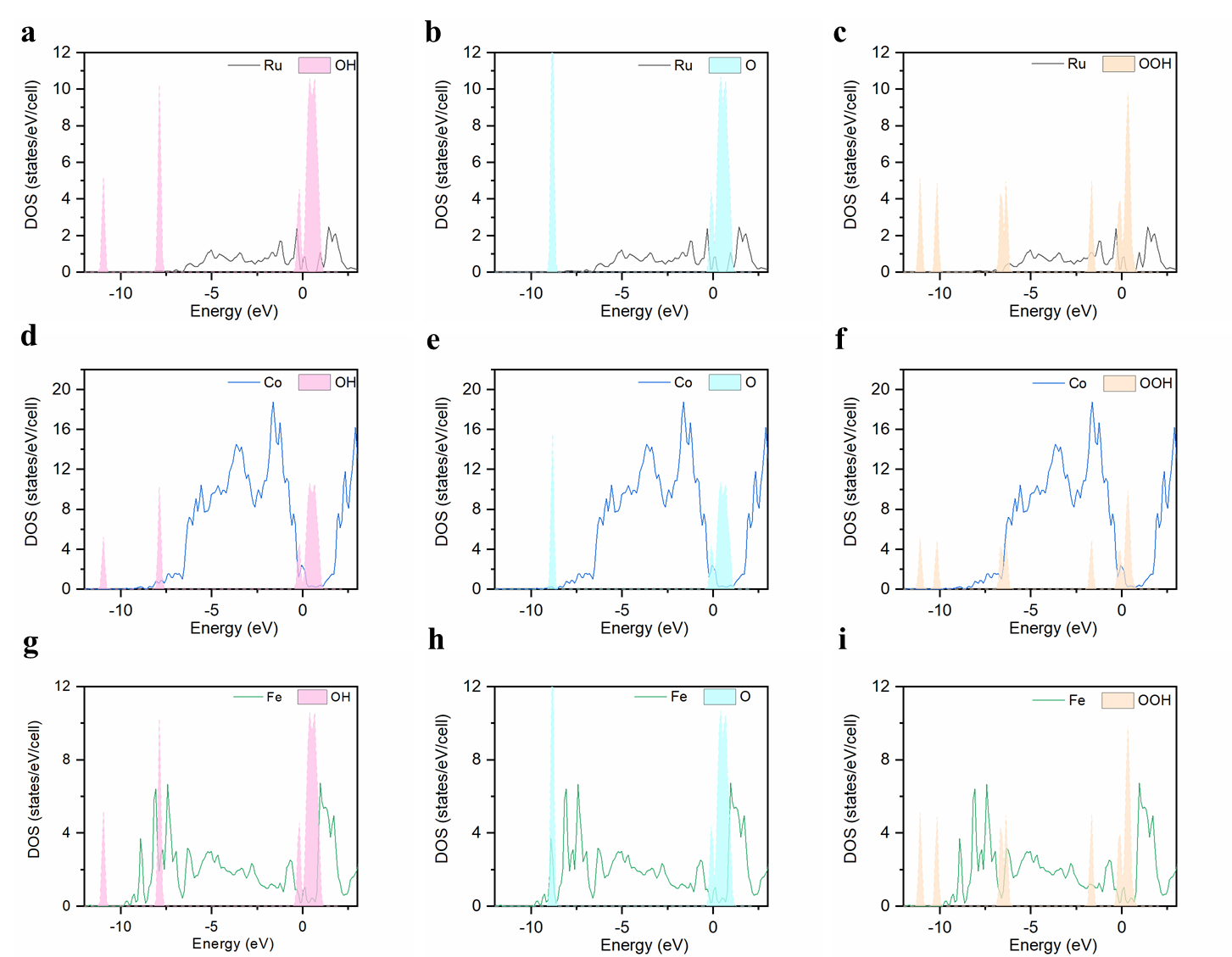
**

**Fig. S20** PDOS diagram of different intermediates in Ru0.51-CoFe-LDH with metal active sites Ru, Co and Fe: (**a**), (**d**) and (**g**) *OH, (**b**), (**e**) and (**h**) *O, (**c**), (**f**) and (**i**) *OOH


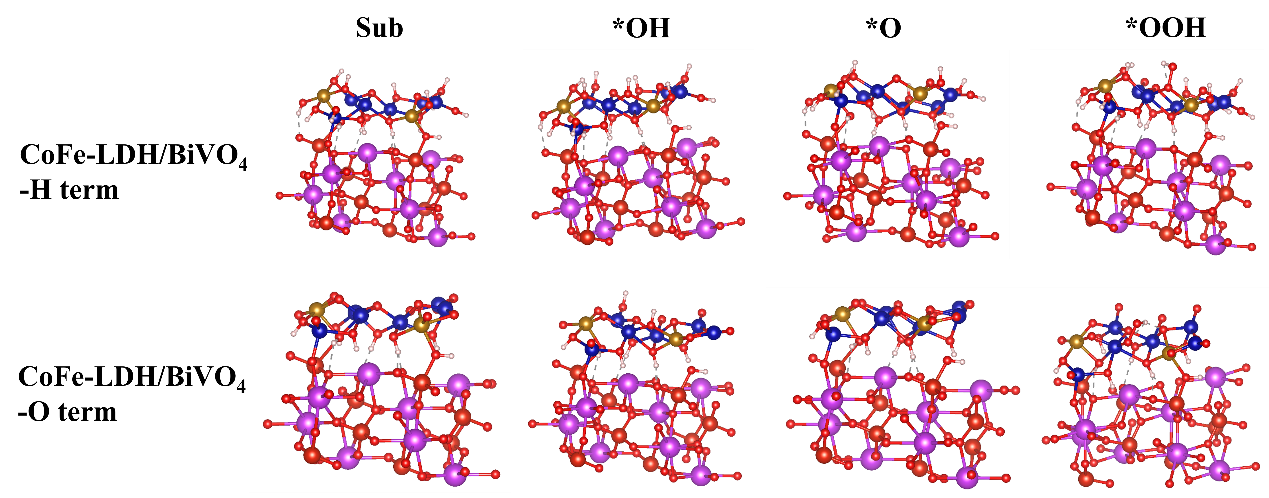


**Fig. S21** Four electron step reaction pathway models for CoFe-LDH/BiVO_4_





**Fig. S22** OER Gibbs free energy diagram of CoFe-LDH/BiVO_4_ with O termination at 0 V

**Table S2** Zeta potential value of BiVO_4_, CoFe-LDH and Ru_0.51_-CoFe-LDH

| Samples | Z_1_ (mV) | Z_2_ (mV) | Z_3_ (mV) | Average (mV) |
| --- | --- | --- | --- | --- |
| BiVO_4_ | -19.4 | -20.3 | -20.5 | -20.1 |
| CoFe-LDH | 25.1 | 24.6 | 25.5 | 25.1 |
| Ru_0.51_-CoFe-LDH | 33.0 | 33.1 | 32.0 | 32.7 |

**Table S3** The peak areas of different forms of oxygen were obtained by fitting the XPS O 1s peaks of BiVO_4_, CoFe-LDH/BiVO_4_ and Ru_0.51_-CoFe-LDH/BiVO_4_ photoanodes

| Samples | XPS O 1s peak area (percentage content) | | |
| --- | --- | --- | --- |
|  | M-O-M | M-OH | O-O |
| BiVO_4_ | 56381 (78.7%) | 13726 (19.1%) | 1573 (2.2%) |
| CoFe-LDH/BiVO_4_ | 43577 (49.4%) | 33302 (37.7%) | 11361(12.9%) |
| Ru_0.51_-CoFe-LDH/BiVO_4_ | 42788 (50.0%) | 36343 (42.5%) | 6382 (7.5%) |

**Table S4** EXAFS fitting parameters at the **Ru K**-edge for various samples

| Sample | Path | CN | R (Å) | σ^2^ (Å^2^) | ΔE (eV) | R factor |
| --- | --- | --- | --- | --- | --- | --- |
| Ru_0.51_-CoFe-LDH | Ru-O | 4.0±0.2 | 1.99±0.02 | 0.0041 | 0.3 | 0.0085 |
| Ru foil | Ru-Ru | 12* | 2.67±0.01 | 0.0058 | -2.3 | 0.0098 |

*^a^CN*, coordination number; *^b^R*, distance between absorber and backscatter atoms; *^c^σ*^2^, Debye-Waller factor to account for both thermal and structural disorders; *^d^ΔE*_0_, inner potential correction; *R* factor indicates the goodness of the fit. Fitting range: 3.0 ≤ *k* (/Å) ≤ 12 and 1.0 ≤ *R* (Å) ≤ 3.0 (Ru1); 3.0 ≤ *k* (/Å) ≤ 12 and 1.0 ≤ *R* (Å) ≤ 3.0 (Ru foil) A reasonable range of EXAFS fitting parameters: 0.700 < *Ѕ*_0_^2^ < 1.000; *CN >* 0; *σ*^2^ > 0 Å^2^; |Δ*E*_0_| < 10 eV; *R* factor < 0.02.

**Table S5** Comparative literature on BiVO_4_-based photoanodes PEC splitting water

| Photoanode | Electrolyte | *J* (mA cm^-1^) at 1.23 V vs. RHE | Ref. |
| --- | --- | --- | --- |
| **Ru_0.51_-CoFe-LDH/BiVO_4_** | **0.2 M KPi**  **(pH = 7)** | **4.51 mA cm^-2^** | **This work** |
| Mo:BVO/CoAl-LDH-u | 0.5 M KBi  (pH = 9.5) | 5.8 mA cm^-2^ | [S9] |
| BiVO_4_/NiCo-LDH-Act | 0.5 M Na_2_SO_4_  (pH = 7) | 3.29 mA cm^-2^ | [S10] |
| BiVO_4_@NiFe-LDHs/Ru | 0.5 M KBi  (pH = 9.33) | 4.65 mA cm^-2^ | [S11] |
| CoNi–LDH/BiVO_4_ | 0.5 M phosphate buffer (pH = 7) | 2.40 mA cm^-2^ | [S12] |
| SAs Pt/AC-CoFe/BiVO_4_ | 1 M KBi | 5.14 mA cm^-2^ | [S13] |
| CoFe(C)/BiVO_4_ | 0.1 M potassium borate buffer | 4.3 mA cm^-2^ | [S14] |
| NiFe-LDH/Ni/BiVO_4_ | 0.5 M KBi  (pH = 9.5) | 4.50 mA cm^-2^ | [S15] |
| BiVO_4_/FeOOH/ZnFe-LDH | 1 M Na_2_SO_4_  (pH = 7) | 4.92 mA cm^-2^ | [S16] |
| H-CoAl-LDH/BiVO_4_ | 0.5 M Na_2_SO_4_  (pH = 7.35) | 3.5 mA cm^-2^ | [S17] |
| BiVO_4_/NiFe-CE | 0.5 M Na_2_SO_4_ | 4.03 mA cm^-2^ | [S18] |
| BiVO_4_/CoMn-LDH | 0.5 M Kpi  (pH = 7) | 2.69 mA cm^-2^ | [S19] |
| ZnCoV–LDH/BiVO_4_ | 0.1 M sodium borate (NaBi, pH 9.4) | 2.7 mA cm^-2^ | [S20] |
| NiCo-LDH/BiVO_4_ | 0.5 M Na_2_SO_4_  (pH ≈ 7.3) | 3.4 mA cm^-2^ | [S21] |
| WCoFe/BiVO_4_ | 0.2 M KPi  (pH = 7) | 4.35 mA cm^-2^ | [S22] |
| NiFeY-LDH/BiVO_4_ | 1 M KBi  (pH = 9.5) | 5.2 mA cm^-2^ | [S23] |
| BiVO_4_/rGO/NiFe-LDH | 1 M KBi  (pH = 9.33) | 3.26 mA cm^-2^ | [S24] |
| BiV_0.97_Mo_0.03_O_4_/NiFe-LDH | 0.1 M Na_2_SO_4_  (pH = 7) | 1.58 mA cm^-2^ | [S25] |
| FeOOH/Ni-N_4_-O/BiVO_4_ | 0.5 M KBi  (pH = 9.3) | 6.0 mA cm^-2^ | [S26] |
| BiVO_4_@NiO−Ir | 0.5 M Na_2_SO_4_  (pH = 7) | 4.33 mA cm^-2^ | [S27] |

**Table S6** Summary of EIS fitting parameters for three photoanodes

| Samples | R_s_/Ω | CPE-T | CPE-P | R_ct_/Ω |
| --- | --- | --- | --- | --- |
| BiVO_4_ | 27.06 | 4.865×10^-5^ | 0.8970 | **564.8** |
| CoFe-LDH/BiVO_4_ | 25.47 | 4.507×10^-5^ | 0.8781 | **481.6** |
| Ru_0.51_-CoFe-LDH/BiVO_4_ | 30.15 | 5.086×10^-5^ | 0.9128 | **272.7** |

# Supplemenary References

1. P. Li, M. Wang, X. Duan, L. Zheng, X. Cheng et al., Boosting oxygen evolution of single-atomic ruthenium through electronic coupling with cobalt-iron layered double hydroxides. Nat. Commun. **10**, 1711 (2019). <https://doi.org/10.1038/s41467-019-09666-0>
2. L. Gao, H. Sun, H. Sun, Y. Wang, Y. Li et al., Embedding Ru single atom catalysts on Co_3_O_4_ for efficient hydrazine oxidation and direct hydrazine fuel cells. Appl. Catal. B Environ. Energy **358**, 124287 (2024). <https://doi.org/10.1016/j.apcatb.2024.124287>
3. S. Grimme, J. Antony, S. Ehrlich, H. Krieg, A consistent and accurate *ab initio* parametrization of density functional dispersion correction (DFT-D) for the 94 elements H-Pu. J. Chem. Phys. **132**(15), 154104 (2010). <https://doi.org/10.1063/1.3382344>
4. S. Grimme, S. Ehrlich, L. Goerigk, Effect of the damping function in dispersion corrected density functional theory. J. Comput. Chem. **32**(7), 1456–1465 (2011). <https://doi.org/10.1002/jcc.21759>
5. K. Mathew, R. Sundararaman, K. Letchworth-Weaver, T.A. Arias, R.G. Hennig, Implicit solvation model for density-functional study of nanocrystal surfaces and reaction pathways. J. Chem. Phys. **140**(8), 084106 (2014). <https://doi.org/10.1063/1.4865107>
6. B. Liu, X. Wang, Y. Zhang, L. Xu, T. Wang et al., A BiVO4 photoanode with a VO*_x_* layer bearing oxygen vacancies offers improved charge transfer and oxygen evolution kinetics in photoelectrochemical water splitting. Angew. Chem. **135**(10), e202217346 (2023). <https://doi.org/10.1002/ange.202217346>
7. Q. Sun, T. Cheng, Z. Liu, L. Qi, A cobalt silicate modified BiVO4 photoanode for efficient solar water oxidation. Appl. Catal. B Environ. **277**, 119189 (2020). <https://doi.org/10.1016/j.apcatb.2020.119189>
8. B. Baral, D.P. Sahoo, K. Parida, Discriminatory{040}-reduction facet/Ag(0) Schottky barrier coupled{040/110}-BiVO(4)@Ag@CoAl-LDH Z-scheme isotype heterostructure. Inorg. Chem. **60**(3), 1698–1715 (2021). <https://doi.org/10.1021/acs.inorgchem.0c03210>
9. Y. Zhong, C. Wu, X. Jia, S. Sun, D. Chen et al., Coupling of self-healing atomic layer CoAl-LDH onto Mo: BiVO4 photoanode for fast surface charge transfer toward stable and high-performance water splitting. Chem. Eng. J. **465**, 142893 (2023). <https://doi.org/10.1016/j.cej.2023.142893>
10. Y. Miao, Z. Li, Y. Song, K. Fan, J. Guo et al., Surface active oxygen engineering of photoanodes to boost photoelectrochemical water and alcohol oxidation coupled with hydrogen production. Appl. Catal. B Environ. **323**, 122147 (2023). <https://doi.org/10.1016/j.apcatb.2022.122147>
11. Y. Sun, H. Li, Y. Hu, J. Wang, A. Li et al., Single-atomic ruthenium coupling with NiFe layered double hydroxide *in situ* growth on BiVO_4_ photoanode for boosting photoelectrochemical water splitting. Appl. Catal. B Environ. **340**, 123269 (2024). <https://doi.org/10.1016/j.apcatb.2023.123269>
12. T.N. Jahangir, A.Z. Khan, T.A. Kandiel, B.M. El Ali, Insights into the charge transfer kinetics in BiVO_4_ photoanodes modified with transition metal-based oxygen evolution electrocatalysts. Catal. Today **413**, 113918 (2023). <https://doi.org/10.1016/j.cattod.2022.09.024>
13. M. Gao, N.T. Nguyen, R.-T. Gao, X. Liu, X. Zhang et al., Engineering single Pt Atoms on hybrid amorphous/crystalline CoFe layered double hydroxide accelerates the charge transfer for solar water splitting. Appl. Catal. B Environ. **336**, 122920 (2023). <https://doi.org/10.1016/j.apcatb.2023.122920>
14. P. Wei, Y. Wen, K. Lin, X. Li, Turning off the “shunt channel” by coating with CoFe layered double hydroxide nanocrystals for efficient photoelectrocatalytic water splitting. Inorg. Chem. Front. **9**(18), 4685–4694 (2022). <https://doi.org/10.1039/d2qi00760f>
15. X. Wang, Y. Lei, Y. Gao, X. Yun, Z. Wang et al., Multi-function of the Ni interlayer in the design of a BiVO_4_-based photoanode for photoelectrochemical water splitting. ACS Appl. Mater. Interfaces **14**(43), 48682–48693 (2022). <https://doi.org/10.1021/acsami.2c13897>
16. C. Liu, Y. Zhang, G. Yin, T. Shi, Y. Zhang et al., Fabricating BiVO4/FeOOH/ZnFe-LDH hierarchical core–shell nanorod arrays for visible-light-driven photoelectrochemical water oxidation. Inorg. Chem. Front. **9**(24), 6431–6440 (2022). <https://doi.org/10.1039/d2qi01165d>
17. P. Yue, H. She, L. Zhang, B. Niu, R. Lian et al., Super-hydrophilic CoAl-LDH on BiVO4 for enhanced photoelectrochemical water oxidation activity. Appl. Catal. B Environ. **286**, 119875 (2021). <https://doi.org/10.1016/j.apcatb.2021.119875>
18. D.A. Reddy, K.A.J. Reddy, M. Gopannagari, Y. Kim, A.P. Rangappa et al., Exposure of NiFe-LDH active sites by cation–exchange to promote photoelectrochemical water splitting performance. Appl. Surf. Sci. **570**, 151134 (2021). <https://doi.org/10.1016/j.apsusc.2021.151134>
19. F. Zhao, N. Li, Y. Wu, X. Wen, Q. Zhao et al., BiVO4 photoanode decorated with cobalt-manganese layered double hydroxides for enhanced photoelectrochemical water oxidation. Int. J. Hydrog. Energy **45**(56), 31902–31912 (2020). <https://doi.org/10.1016/j.ijhydene.2020.08.224>
20. T.-G. Vo, K.-F. Chang, C.-Y. Chiang, Valence modulation on zinc-cobalt-vanadium layered double hydroxide nanosheet for accelerating BiVO4 photoelectrochemical water oxidation. J. Catal. **391**, 336–345 (2020). <https://doi.org/10.1016/j.jcat.2020.09.001>
21. H. She, P. Yue, X. Ma, J. Huang, L. Wang et al., Fabrication of BiVO4 photoanode cocatalyzed with NiCo-layered double hydroxide for enhanced photoactivity of water oxidation. Appl. Catal. B Environ. **263**, 118280 (2020). <https://doi.org/10.1016/j.apcatb.2019.118280>
22. W. Li, L. Du, Q. Liu, Y. Liu, D. Li et al., Trimetallic oxyhydroxide modified 3D coral-like BiVO4 photoanode for efficient solar water splitting. Chem. Eng. J. **384**, 123323 (2020). <https://doi.org/10.1016/j.cej.2019.123323>
23. D. He, R.-T. Gao, S. Liu, M. Sun, X. Liu et al., Yttrium-induced regulation of electron density in NiFe layered double hydroxides yields stable solar water splitting. ACS Catal. **10**(18), 10570–10576 (2020). <https://doi.org/10.1021/acscatal.0c03272>
24. H. Chen, S. Wang, J. Wu, X. Zhang, J. Zhang et al., Identifying dual functions of rGO in a BiVO4/rGO/NiFe-layered double hydroxide photoanode for efficient photoelectrochemical water splitting. J. Mater. Chem. A **8**(26), 13231–13240 (2020). <https://doi.org/10.1039/d0ta04572a>
25. J. Guo, X. Yang, S. Bai, X. Xiang, R. Luo et al., Effect of Mo doping and NiFe-LDH cocatalyst on PEC water oxidation efficiency. J. Colloid Interface Sci. **540**, 9–19 (2019). <https://doi.org/10.1016/j.jcis.2018.12.069>
26. X. Zhang, P. Zhai, Y. Zhang, Y. Wu, C. Wang et al., Engineering single-atomic Ni-N(4)-O sites on semiconductor photoanodes for high-performance photoelectrochemical water splitting. J. Am. Chem. Soc. **143**(49), 20657–20669 (2021). <https://doi.org/10.1021/jacs.1c07391>
27. D.A. Reddy, Y. Kim, K.A.J. Reddy, M. Gopannagari, A.P. Rangappa et al., Boosting water oxidation performance of BiVO_4_ photoanode by vertically stacked NiO nanosheets coupled with atomically dispersed iridium sites. ACS Appl. Energy Mater. **4**(10), 11353–11366 (2021). <https://doi.org/10.1021/acsaem.1c02181>
